# Mechanochemistry: A Green Approach in the Preparation of Pharmaceutical Cocrystals

**DOI:** 10.3390/pharmaceutics13060790

**Published:** 2021-05-25

**Authors:** Mizraín Solares-Briones, Guadalupe Coyote-Dotor, José C. Páez-Franco, Miriam R. Zermeño-Ortega, Carmen Myriam de la O Contreras, Daniel Canseco-González, Alcives Avila-Sorrosa, David Morales-Morales, Juan M. Germán-Acacio

**Affiliations:** 1 Red de Apoyo a la Investigación, Coordinación de la Investigación Científica-UNAM, Instituto Nacional de Ciencias Médicas y Nutrición SZ, Ciudad de México, C.P. 14000, Mexico; misolb13@gmail.com (M.S.-B.); ibtcoyotedg@gmail.com (G.C.-D.); paez@cic.unam.mx (J.C.P.-F.); 2 Facultad de Ciencias Químicas, Universidad Autónoma de Chihuahua, Circuito Universitario No. 1, Nuevo Campus Universitario, Apdo. Postal 1552, Chihuahua, C.P. 31125, Mexico; mrzermeno@uach.mx (M.R.Z.-O.); cdelao@uach.mx (C.M.d.l.OC.); 3 CONACYT-Laboratorio Nacional de Investigación y Servicio Agroalimentario y Forestal, Universidad Autónoma de Chapingo, Texcoco de Mora, C.P. 56230, Mexico; jdcanseco@conacyt.mx; 4 Instituto Politécnico Nacional, Escuela Nacional de Ciencias Biológicas, Departamento de Química Orgánica, Carpio y Plan de Ayala S/N, Colonia Santo Tomás, Ciudad de México, C.P. 11340, Mexico; aavilas@ipn.mx; 5 Instituto de Química, Universidad Nacional Autónoma de México, Circuito Exterior, Ciudad Universitaria, Ciudad de México, C.P. 04510, Mexico

**Keywords:** mechanochemistry, green reactions, pharmaceutical cocrystals

## Abstract

Mechanochemistry is considered an alternative attractive greener approach to prepare diverse molecular compounds and has become an important synthetic tool in different fields (e.g., physics, chemistry, and material science) since is considered an ecofriendly procedure that can be carried out under solvent free conditions or in the presence of minimal quantities of solvent (catalytic amounts). Being able to substitute, in many cases, classical solution reactions often requiring significant amounts of solvents. These sustainable methods have had an enormous impact on a great variety of chemistry fields, including catalysis, organic synthesis, metal complexes formation, preparation of multicomponent pharmaceutical solid forms, etc. In this sense, we are interested in highlighting the advantages of mechanochemical methods on the obtaining of pharmaceutical cocrystals. Hence, in this review, we describe and discuss the relevance of mechanochemical procedures in the formation of multicomponent solid forms focusing on pharmaceutical cocrystals. Additionally, at the end of this paper, we collect a chronological survey of the most representative scientific papers reporting the mechanochemical synthesis of cocrystals.

## 1. Introduction

Grant et al., reported that the intrinsic activity of a drug is immensely influenced by its molecular structure and its supramolecular arrangement [1]. Most of the 90% of marketed pharmaceutical products are sold as solid forms: tablets, capsules, suppositories, etc. [2]. The drug efficacy largely depends on their physicochemical and materials properties in the solid state, because its performance can be affected by different crystalline states [1]. It is said that approximately 80% of all drug molecules exhibit polymorphism [3]. Is for this reason that pharmaceutical companies invest enormous amounts of money in the development of high-throughput experimental technologies to determine the crystalline diversity of a drug [4,5,6,7,8]. Crystallization methods in the pharmaceutical industry represent one of the most recurrent practices in the separation and purification of an active pharmaceutical ingredient (API). After partial or total synthesis of an API, crystallization is considered the most efficient purification process compared with liquid-liquid extraction or chromatography [9]. Thus, in to ensure purity of the crystal, shape, and size of the crystal distribution, and the crystal habit of a drug, the proper choice of solvent plays an important role [10,11].

Drugs may exhibit different solid forms, and this diversity may modify their physicochemical and biological properties, such as melting point, thermal stability, solubility and dissolution rate, and bioavailability [12,13,14,15,16,17,18]. For instance, nearly 40% of the approved drugs and 90% of the developmental pipeline drugs show low solubility [17,19,20]. The determination of the different solid/crystalline forms adopted by an API during its development process is fundamental since it may have a great impact in its scale-up, formulation, and clinical trials; and in some cases, may avoid patent litigations among the pharmaceutical companies [16,18,21].

Drug efficacy depends at least on three pharmaceutical and pharmacological factors: potency (dose), solubility, and permeability [22,23,24]. By itself, potency is an intrinsic property of the molecule and is difficult to manipulate, however, solubility and permeability can be modulated. The biopharmaceutics classification system (BCS) is a guidance to categorize drugs on the basis of its aqueous solubility and intestinal permeability [25]. This protocol classifies drugs in four groups: Class I (high solubility and permeability); Class II (low solubility and high permeability); Class III (high solubility and low permeability); and Class IV (low solubility and permeability) [26,27].

The modulation of the drug permeability can be reached by various methods: (1) metabolism inhibitors [28], (2) ion-pairing and complexing agents [29], (3) lipid and surfactant adjuvants [30], and (4) inhibitors of secretory proteins [31]. In addition, many approaches are employed to enhance low-solubility drug issues, such as: chemical modifications (prodrugs or preparation of salts) [32,33] and development of new dosage forms (cyclodextrin complexes, lipid formulations, drug-carrier formulations: polymers, surfactants, carbohydrates, dendrimers, etc.) [34,35,36,37,38]. The restrictions found in chemical modifications are that prodrug formation may represent an elevated-cost process subsequent to the chemical derivatization and the structural modification can affect the toxicological profile of the molecule [39]. Salt formation largely depends on the ionizable functional groups that the drug has, otherwise the process is difficult and furthermore this procedure sometimes can modify the properties of the parent drug [40]. On the other hand, the limitations encountered in new dosage forms using cyclodextrin complexes are: size compatibility cavity/drug, reversibility of complex, and sometimes cyclodextrins have been related with toxic reactions, however, apparently these issues were more likely due impurities [41]. Additionally, lipid formulations only admit the entrapment of lipophilic drugs, and drugs with high melting point and log *p* > 2 are poor candidates [42]. Recurrent hurdles found in drug-carrier formulations include: (1) carriers used frequently are hygroscopic absorbing water destabilizing the system, (2) sometimes high amounts of carrier are required to ensure the molecular mixing, (3) scale-up problems, and (4) thermodynamic instability [35,43].

**Figure 1:**
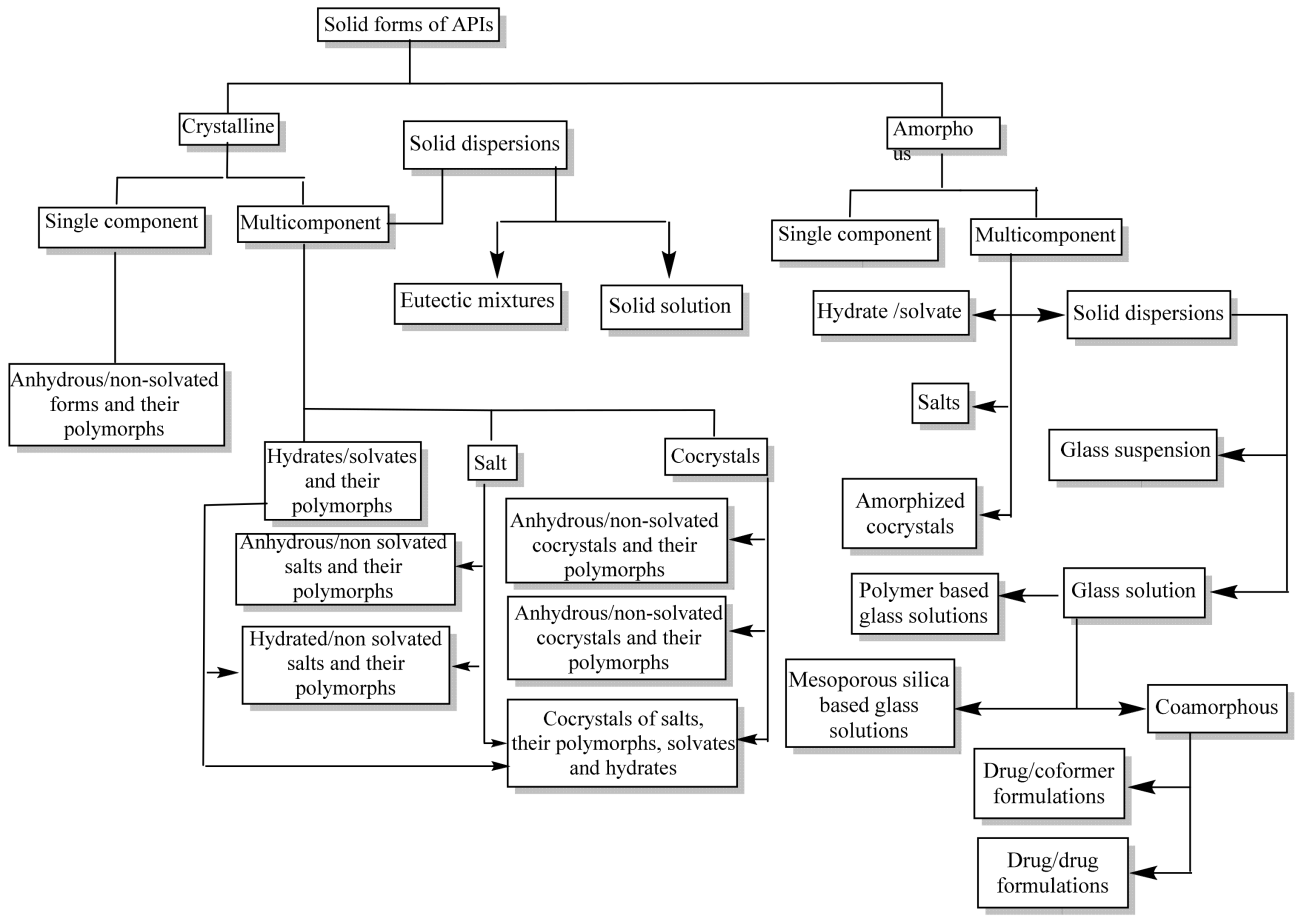
Classification of an API depending on its scale range-order periodicity and composition. Adapted with permission from [15], Elsevier, 2017.

The formation of multicomponent solid forms is another important approach used for the modulation of drug solubility and dissolution rate properties [44,45,46,47,48,49,50,51,52,53]. This approach may offer important drug property improvements (physicochemical and biological), modifying the molecular conformations and intermolecular interactions of the parent API due to incorporation of a second molecular agent (coformer or drug), without affecting its intrinsic activities [48,54,55]. All drugs with solubility issues are candidates to form multicomponent solid forms, contrary to some of the methods mentioned above i.e., salt formation, cyclodextrin complexes, and lipid formulation. Thus, the pharmaceutical industry and the scientific community have proposed an ample view of how we must classify the different solid forms observed in an API, Figure 1. This holistic view is based on scale range-order periodicity and composition diversity, from single to multicomponent forms [14,15,18].

Pharmaceutical multicomponent solid systems can solidify in various forms: cocrystals [56,57,58], salts [59,60,61,62], solid eutectic compositions [50,51,63,64], coamorphous [52,53,64,65,66,67], polymorphs of cocrystals [68,69,70,71,72,73,74,75], etc.

Pharmaceutical solid forms: cocrystals/multicomponent salts [76,77], 22 eutectic mixtures [50,51,63,64,78], coamorphous [53,64] can be prepared by diverse methods, however, recently solvent free solid-state processes have gained a lot of interest, since they can take place even in the absence or with the minimal amounts of solvent (catalytic amounts).

These free solvent reactions are different compared with the traditional solution reactions (dissolving, heating, and stirring). Mechanochemistry, by itself is an important synthetic tool branch of solid state-chemistry [79,80]. Mechanochemistry is related mainly with the chemical transformation of matter induced by mechanical energy by grinding or milling. Mechanochemistry has emerged as an important green tool of synthesis in diverse fields (e.g., physics, chemistry, material science). Recently, mechanochemistry has had an important impact in a great variety of synthetic fields of the chemistry, including catalysis [81,82,83,84,85,86], synthesis of organometallic compounds [87,88,89,90,91], organic synthesis [83,92,93,94,95,96,97], metal complexes preparation [98,99,100], main-group elements [101,102], porous metal-organic frameworks (MOFs) [81,99,103,104,105,106,107], polymers [108,109,110], fullerenes [111], multicomponent pharmaceutical materials [53,81,99,105,112,113,114,115,116,117], etc. Thus, the main focus of this review is to highlight the benefits of mechanochemical reactions in the preparation of pharmaceutical cocrystals. First, the paper will provide the reader with the definition of mechanochemistry, historical aspects, comparisons with other sustainable techniques, etc. Then, the main characteristics of cocrystals, definition, their diverse analytical methods to characterize, diverse synthetic methods, etc., will be described. Finally, a chronological summary of the most relevant papers concerning with their mechanochemical preparation will be presented.

## 2. Mechanochemistry

### 2.1. Definitions, Relevant Historical Aspects, and Applications of Mechanochemistry

The old belief that the success of a chemical reaction depends of the presence of large amounts of a solvent is no longer valid [118]. Recently, enormous interest has been focused on the study of ecofriendly and sustainable reactions, with the aim to perform them under solventless conditions or at least with a minimal utilization of solvents. These kinds of reactions fall in the realm of Green Chemistry, prioritizing high yields and mild conditions [119]. Green Chemistry is a discipline prone to reduce the utilization of environmentally noxious materials and energetic resources. Green Chemistry promotes the development of benign reactions; limiting the use of solvents and finding the optimization of all resources employed (materials, reactants, reagents, solvents, and energy requirements). Green Chemistry is ruled by twelve lineaments [120,121].

**Figure 2:**
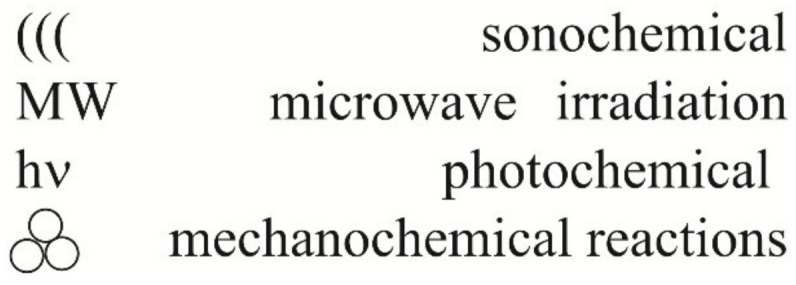
Symbolic representations of the different solid state-chemistry synthetic methods. Adapted with permission from [101], Royal Society of Chemistry, 2019.

On the other hand, solid state-chemistry explores the reactivity of the substances in solid state, through different synthetic methods such as: [92] microwave irradiation [122,123], ultrasound (sonochemistry) [123,124,125], photochemistry [126,127,128,129], mechanochemistry [105,130]. A symbolic representation of these different synthetic methods is depicted in Figure 2 [101].

Mechanochemistry is a term related with the chemical reactivity promoted by diverse mechanical stimulus, (typically friction, impact, collision, grinding). A mechanochemical reaction is defined by the IUPAC as: “a chemical reaction that is induced by the direct absorption of mechanical energy” [131]. Mechanochemistry through the time have received several definitions [132]. Wilhelm Ostwald classified overall the chemistry in diverse fields: thermochemistry, electrochemistry, photochemistry, etc., depending on the energy input [105]. From this classification, it should be noted that Wilhelm Ostwald separated the mechanochemistry as a unique branch of chemistry [133,134]. Later, Walther Nernst (Ostwald’s student) supported this classification [105,135]. However, according to Baláž, the current accepted definition is that of Heinicke that establishes that “mechanochemistry is a branch of chemistry which is concerned with the chemical and physicochemical transformations of substances in all states of aggregation produced by the effect of mechanical energy” [132].

**Figure 3:**
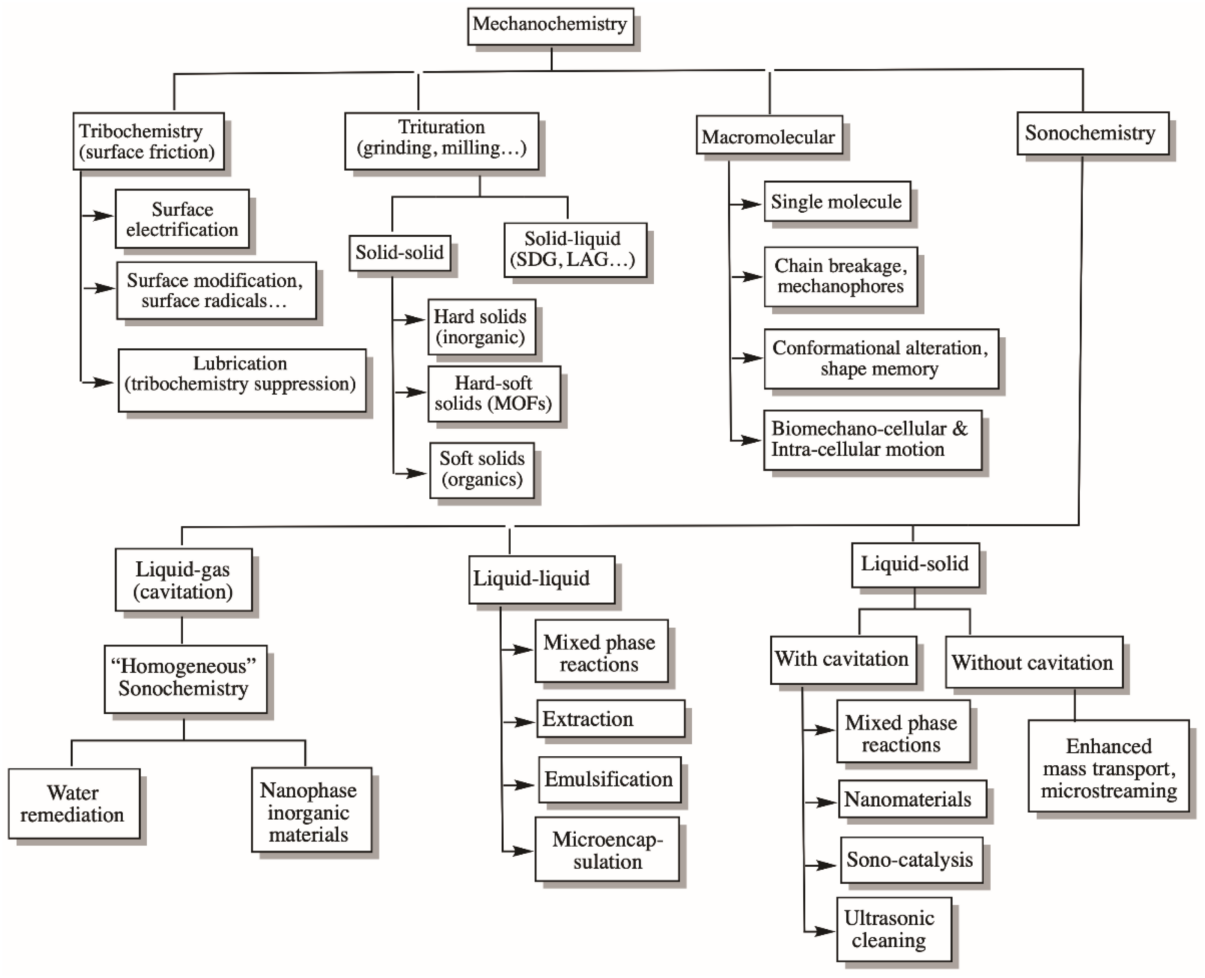
Different branches derived from the mechanochemistry. Adapted with permission from [80], Royal Society of Chemistry, 2014. SDG: solvent drop-grinding; LAG: liquid-assisted grinding; MOFs: metal-organic frameworks.

Sometimes tribochemistry (*vide infra*) is confused with mechanochemistry, however, by itself the former term is a branch of the latter, Figure 3 [80]. Tribochemistry (friction, *tribos*), per se is related with chemical reactions occurred between two solid surfaces, within the lubricating material, or between the lubricant and solid surfaces [136,137]. Thus, in general, mechanochemistry it should be considered into four areas: tribochemistry (the chemistry of surfaces in contact), trituration (chemistry induced by grinding and milling), macromolecular mechanochemistry (from breakage of polymer chains to molecular motors and biological motion), and sonochemistry (the chemistry generated from the mechanical consequences of sound), Figure 3 [80].

Mechanochemistry can be dated back to the prehistoric times [138], Greek ages [Theophrastus of Ephesus (371–286 B.C.)] [139,140] and at the period between the 19th and the early 20th century [105,113,138,141,142,143]. Particularly in the 19th and 20th centuries, the pioneering work of Carey Lea (according with Takacs the first mechanochemist) [134,141,144,145] and Faraday can be highlighted [142,143]. For an excellent detailed description of the development of mechanochemistry through the history, “The historical development of mechanochemistry” must be consulted [143].

The recent interest in mechanochemical methods is due to diverse advantages compared with the classical-based dissolution reactions. According to Etter: “the absence or at least the minimal usage of solvents during the course of the reactions, often leads by mechanochemical methods to the phase similar to that obtained by solution crystal growth”, suggesting that the presence of large amounts of solvent is not necessary for the formation of a new phase [105,146,147,148,149]. Mechanochemical methods are preferred to be used when the product is not accessible via conventional reactions (classical dissolution) [150,151,152,153,154]. In addition, mechanochemical processes can provide control over the polymorphic outcome, sometimes not obtained in solution [155,156,157,158,159,160,161,162]. In addition, yields can be improved and reactions may proceed faster than in solution [98,163,164,165,166]. Furthermore, improved stereochemical control and selectivity can be achieved [167,168]. Additionally, reactions can be carried out to completion, with additional purification steps not necessary [169,170]. Some reactions exhibit a reduced energy consumption due to efficient energy transfer in the mixture reaction (e.g., planetary ball-milling) [171].

Apparently, the first paper describing the formation of a cocrystal using a mechanochemical reaction was reported in 1893 [172]. In this paper was informed the preparation of quinhydrone cocrystals grinding equimolar amounts of *p*-benzoquinone and hydroquinone. After this, the reports of Curtin [173] and Etter [174,175] were stablished as great contributions to the development in the preparation of cocrystals by mechanochemical procedures.

Kaupp indicated that milling, grinding, shearing, kneading, stirring, pulling, and cutting do not constitute part of the mechanochemistry if this stimulus does not produce a bond breaking [79]. The term mechanochemistry is usually ligated with the rupture and formation of covalent bonds [140], but the “non-covalent mechanochemistry”, ref. [176] is part of interest in the preparation of multicomponent pharmaceutical solid forms. Grinding two chemical substances lead to the modification of the intermolecular interactions altering the solid-state properties of the new solid form. The definition of what is a cocrystal will be discussed in Section 3.1.

### 2.2. Different Mechanochemical Apparatuses

Grinding may be so simple as manually grinding two chemical reagents in a mortar and pestle. However, it should be noted, that grinding and milling are different techniques, since grinding is the mechanochemical procedure working in a mortar and milling is carried out in ball mills devices [95]. At the laboratory level, ball-milling is employed rather than grinding, when higher energy input is required, more systematic times of reaction (reproducibility) are needed and better mixing (mass and energy transfer) is sought [92]. Grinding with a mortar depends on the vigor and intensity applied during the process being less systematic and sometimes atmospheric conditions can be an adverse factor [101]. The implementation of automatic devices (milling equipment) can circumvent these hurdles [162]. For a detailed description of the diverse types of milling apparatuses and their characteristics (balls mills to vibratory, planetary, mills, and attritors), proper references must be consulted [132,177,178]. According to Mack, at the laboratory level, there are three typical mills (mixer/shaker mills, planetary, twin screw extrusion) [93]. From which mixing/stirring mills and planetary devices are more accessible to obtain (USD 3000–7000) [93]. For an excellent description of the characteristics and differences associated with mixing/stirring mills and planetary devices, the following relevant references must be consulted [93,100,179,180]. Twin-screw extrusion (TSE) is another mechanochemical tool which can provide large-scale preparation of cocrystals (industrial level). [181,182,183] Additionally, TSE has been used for the scale-up preparation processes in the synthesis of organic compounds [184,185], MOFs [186], and deep eutectic solvents, etc. [187].

### 2.3. Advantages of Reaction Performance among Grinding/Milling and Other Sustainable Methods

**Scheme 1:**
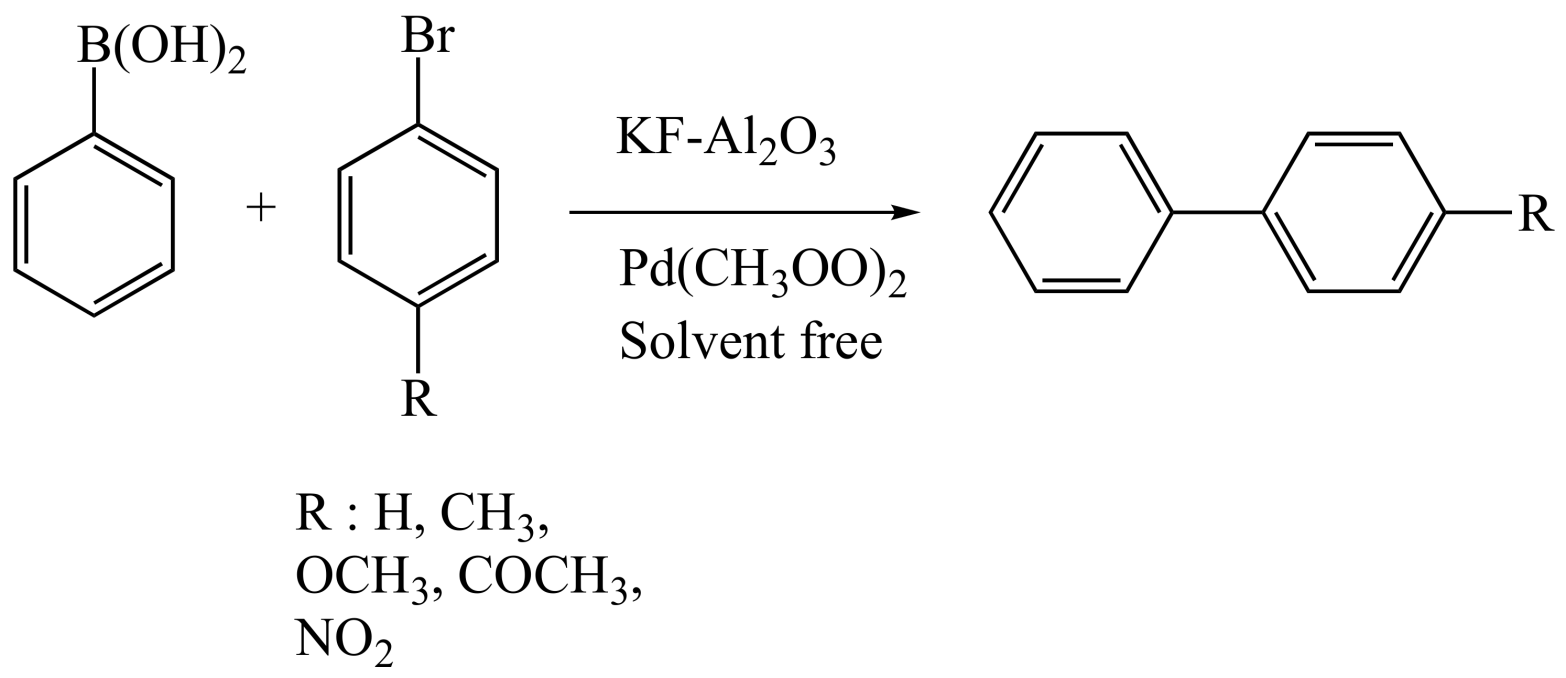
Pd-catalyzed C-C coupling reactions (Suzuki–Miyaura).

Schneider et al., showed the advantages in energy consumption of mechanochemical reactions over other sustainable methods [171]. Thus, in a comparative study of Suzuki-Miyaura reactions (C-C couplings: phenylboronic acid + different aryl bromides) for the production of several biaryls, Scheme 1. Experiments grinding the components with mortar and ball-milling were carried out. Grinding outcomes revealed problems with the reproducibility, not found in ball-milling experiments. In addition, different studies evaluating the energy demand (ball-milling or microwave irradiation) were performed. A third experiment was carried out combining (ball-milling + microwave irradiation, COMB) also seeking to determine the energy consumption. Interestingly, ball-milling experiments required lesser energy demand than microwave assays and the combination of both methods, demonstrating the advantages of mechanochemical synthesis over microwave irradiation. According to the authors, the couplings of the *p*-bromoacetophenone proceed easier that the other substrates due to the fact that the C-Br bond is weaker because of the electron-donating nature of the *p*-substituent, resulting in the best yields in the couplings reactions. Thus, taking this as a model reaction, their yields were evaluated, initially comparing the reaction carried out with a mortar and pestle and in a Pulverisette 7 device (planetary ball mill). From these experiments, it was observed that by grinding in a mortar, the reaction produced yields greater than 50%, but when the milling equipment was used, the performance depended on the rpm used. Producing yields of ~5%, ~70%, and ~89% for 200, 400, and 800 rpm, respectively. A further comparison of the yields employing ball-milling (Pulverisette 7 (at 800 rpm) BM1 or swing/mixer mill BM2), microwave irradiation (MW1: multimode or MW2: mono-mode) or COMB, the following results were obtained: BM1 (89%); BM2 (45%); MW1 (80%); MW2 (70%); COMB (94%), thus showing the best yields to be produced by the COMB method for this particular Suzuki–Miyaura cross coupling reaction. Although the BM1 procedure required lesser energy demand than the other methods.

Another study comparing the efficiency (chemical yield and energy consumption) of ball-milling with different synthetic methods was reported by Thorwirth et al. [188]. The oxidation of primary aromatic amines to azo and azoxy compounds were evaluated by different methods: mechanochemical (planetary and vibratory ball-milling) and solvent-based procedures (microwave, conventional heating, ultrasound). In this case, also the mechanochemical reactions were more efficient in terms of both chemical yield and energy consumption. However, planetary ball-milling was more efficient for a scale-up reactions compared with vibratory milling.

### 2.4. Preparative Conditions of Mechanochemical Methods of Pharmaceutical Cocrystals

**Figure 4:**
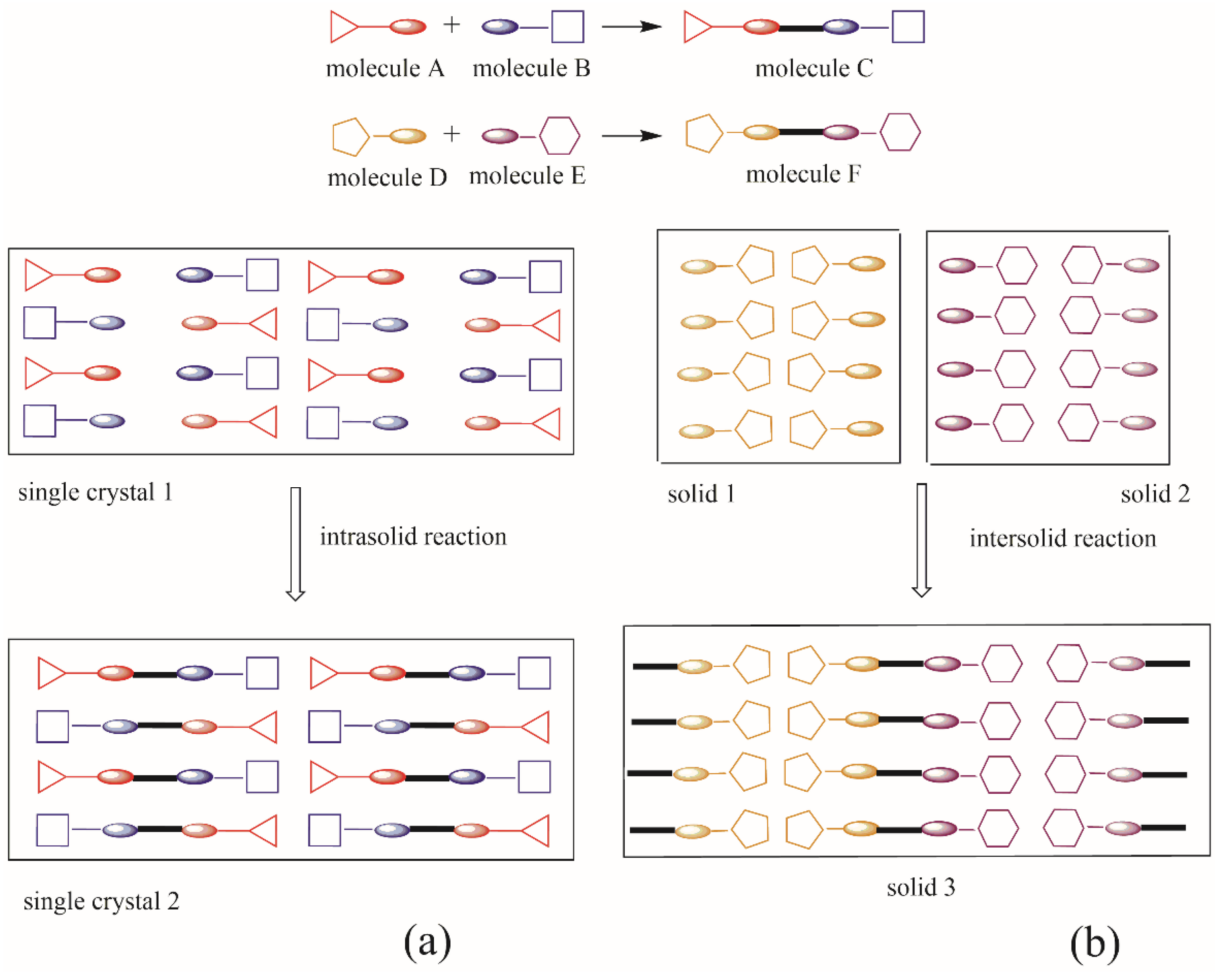
Classification of solid–solid reactions: (**a**) intrasolid and (**b**) intersolid. Adapted with permission from [189], Wiley, 2004.

Braga has classified solid-state reactions as [189,190]:

1. Intrasolid reactions, which proceed amongst molecules within a single-crystal (Topochemical Postulates developed by Schmidt), Figure 4 [191,192,193].
2. Intersolid reactions, which pertain to the reactivity between solids (mechanochemical reactions), Figure 4.
3. Furthermore, Braga added a third category, solid–gas reactions [189,190].

However, Bolm indicates that solventless reactions can be categorized into three groups [194]:

1. Reactions between solids (solid–state reactions).
2. Reactions between solids with intermediate local melting.
3. Reactions with at least one liquid reagent.

One of the persistent problems found in the mechanochemical reactions is the lack of understanding in the mechanisms involved. In this context, mechanochemical reactions can be monitored ex situ [96,195,196] or in situ [84,96,197] using synchrotron XRPD [198,199,200] and Raman spectroscopy [201,202,203], in order to get an insight into the different intermediate steps and pathways involved.

The components involved in a typical mechanochemical reaction can be mixed at least by two different conditions: the “dry” manner denominated neat grinding (NG) or by “wet” conditions [105,112,204]. “Dry” conditions is to grind/mill all the components in total absence of solvent, consequently “wet” conditions are carried out when solvent is added in catalytic amounts. Currently, the accepted term used for “wet” conditions is liquid-assisted grinding (LAG), but in some old literature, is also known as kneading [105]. Although LAG was also formerly known as solvent drop grinding (SDG) [154,155].

To the best of our knowledge, the first paper, the improvement in kinetics of cocrystal formation by grinding under “wet” conditions was reported by Jones et al. [205]. Combining cyclohexane-1,3-cis-5-cis-tricarboxylic acid (CTA, C_6_H_9_(COOH)_3_) in the presence of 4,4′-bipyridine (4,4′-bipy) (1:1) for 1 h under NG conditions lead to the partial formation of the cocrystal. However, the addition of 0.05 mL of methanol in the mixture and grinding by 20 min lead to a significant acceleration of the reaction to achieve the complete formation of the cocrystal. Another multicomponent system explored was CTA + 4,7-phenanthroline (4,7-*phen*) (1:2) under NG. After a long period of time grinding, the reaction only occurred partially. However, if the reaction was carried out in the presence of small amounts of methanol, after 5 min, the reaction proceeded almost quantitatively, although traces of the original reactants were detected. Based on these findings, Jones et al., proved that the kinetics of the formation of cocrystals can be improved by LAG.

Jones and Friščić later showed that outstanding benefits can be obtained by LAG over NG in the preparation of cocrystals [81,82,162,206]. For instance, NG has a slight implication in the molecular change of the course of a mechanochemical reaction, contrary to LAG which confers mobility to the components involved. In this regard, LAG imparts additional degrees of freedom (orientational and conformational) to the molecules affecting the reaction outcome. The empirical parameter η, η = *V*(liquid, μL)/*m* (sample, mg) (volume of solvent, and *m* represents the weights of cocrystal components) helps to assign a scale to distinguish the different conditions in which the reaction is carried out (NG = 0; LAG 0 < η < 2; slurrying 2 < η < 12 and solution synthesis > 12) [81]. This parameter provides an interesting insight of the role of the solvents for the design of experiments that are mediated by the presence of a minimal amount of solvent (catalytic conditions) going through slurrying or liquid phase [206].

The importance of η is illustrated by the work of Jones et al., where he explored the polymorph diversity of the cocrystal caffeine:anthranilic acid (caf:ana) using VALAG (variable amount liquid-assisted grinding) [207]. The screening experiments varying the amount of the solvent used (η parameter range 0.05–0.5) and utilizing 15 solvents of different polarity, revealed that polar solvents (4 of 15): acetonitrile, nitromethane, ethylene glycol, and 1,6-hexanediol yielded one cocrystal polymorphic form. It should be noted that experimental conditions (grinding time, frequency, and ball-to-powder weight ratio) were maintained fixed. However, the utilization of non-polar solvents (11 of 15) led to the formation of two or more different cocrystal polymorphs. Thus, globally, Jones et al., concluded that polymorphic control of the cocrystal can be achieved by the polarity of the solvent used and fixing the η parameter. With this work, Jones demystified the common belief of “one liquid for one specific polymorphic form”.

POLAG (polymer assisted grinding) is another relevant mechanochemical technique for the screening of new pharmaceutical solid forms. As mentioned above in the introduction, for drug-carrier formulations, polymers are used as excipients, mainly to stabilize highly activated molecular structures [43]. In this context, Matzger has explored new crystalline forms using primarily polymers as nucleation inducers for the discovery of new polymorphs [208]. According with Matzger, polymers (are crystallization directors) have the capacity to promote selectively the formation of one polymorph over another [209,210]. Jones et al., continued in this line using POLAG, where by means of mechanochemical reactions using polymers; these materials can direct the formation of specific polymorphs [162,211]. Jones et al., described the preparation of three different cocrystals previously reported in the literature caffeine:citric acid (caf:ca) [212] phenazine and mesaconic acid (phe:ma) [213] and caf:ana [69]) by POLAG. The polymer used as catalyst was polyethylene glycol (PEG) varying its molecular weight from 200 to 10,000.

The cocrystal caf:ca (1:1) can be obtained by LAG (grinding with water) or by NG (only when caf hydrated + ca dehydrated is used). [212] In the second system phe:ma, produced with LAG, a dramatical increase in the cocrystallization process is observed. In addition, for the third system caf:ana, the product can be obtained by NG or LAG, however, LAG promotes cocrystal polymorphism. Additionally, in the system caf:ana, using POLAG yielded a different polymorphic form compared with NG. In general, according with the findings in the three systems, using POLAG (adding PEG) produced similar results to those observed with LAG. However, depending on the amount of polymer added in the mechanochemical procedure, this promotes or inhibits the formation of the cocrystal. For instance, in the system caf:ca, using PEG 10,000 in low amounts (1–5%), the presence of the characteristic peak of caf at 2θ = 12° is noted by powder X ray diffraction (PXRD). However, by increasing the amount of PEG 10,000 to 10% the peak of caf at 2θ = 12° is considerably reduced, but at higher percentages of PEG 10,000 (60%), the intensity of this peak increased again. Experiments using other PEGs (200, 300, 400, 3000, 6000, 10% of polymer), and increasing the times of milling, revealed in the PXRD patterns a considerably reduction of the peaks corresponding to the starting materials. Besides, in general, in the three systems, the polymer chain length did not affect the mechanochemical formation of the cocrystal, revealing POLAG as an excellent method for the control of the powder particle size.

Recently, Germann et al., reported the first in situ PXRD monitored mechanochemical cocrystallization (caffeine:glutaric acid; caf:glu) employing POLAG. [214] The authors introduced the δ parameter for POLAG (equivalent to η parameter used in LAG) [81] to compare the reactions in terms of the amount of grinding additive. In this work, they explored the use of diverse PEGs and different δ values.

**Figure 5:**
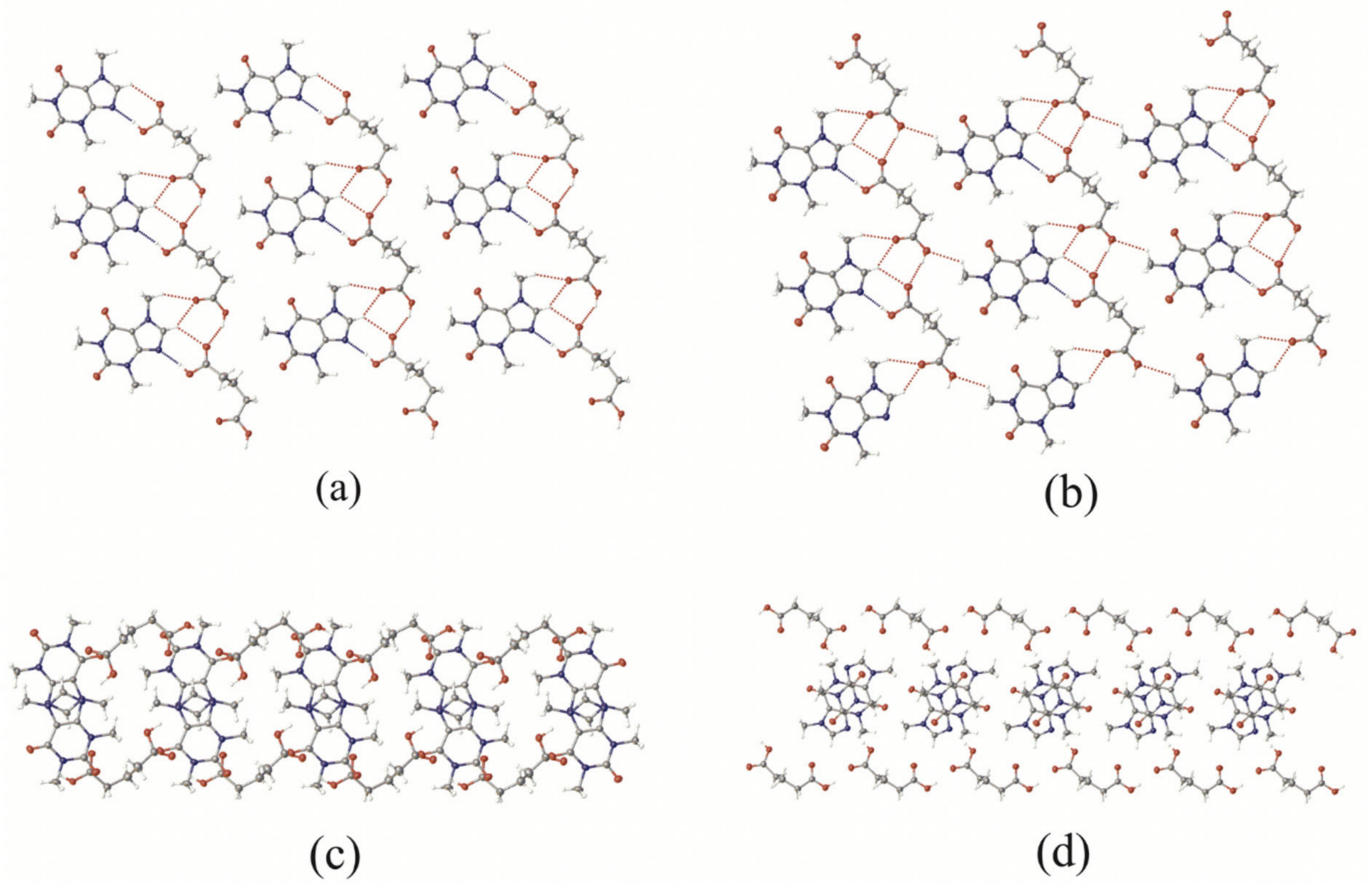
Sheets of caf:glu (1:1) ribbons. (**a**) Form I and (**b**) Form II. Stacked ribbons of caf:glu (1:1): (**c**) Form I and (**d**) Form II. CSD refcodes: Form I; EXUQUJ (*P*2_1_/*c*) and Form II; EXUQUJ01 (Pī).

So far, two forms of the system caf:glu (1:1) have been reported: Form I which is metastable and converts to Form II under high humidity conditions, (CSD refcodes: EXUQUJ (P2_1_/c) and EXUQUJ01 (Pī)), Figure 5. [155,215] However, after the transformation of Form I into Form II, the latter system remains stable for three days before inevitably undergoing conversion to caffeine hydrate. Both forms of caf:glu 1:1 (P2_1_/c or Pī) can be prepared by slow evaporation techniques or NG and LAG [155]. By using NG, Form I can be exclusively produced, but by using LAG, both forms can be obtained. Form I is obtained by LAG using non-polar solvents (hexane or heptane) whilst Form II was formed preferably employing polar solvents (acetonitrile or dichloromethane).

The first milling experiments carried out in this work used NG and LAG (acetonitrile, η = 0.05 mL·mg^−1^). In NG, Form I was formed in the first 7 min of reaction. LAG experiments were faster than NG reactions. In LAG, firstly, Form I was formed as an intermediate, but subsequently, Form II appeared as the final product. LAG experiments adjusting η to higher values (0.12 and 0.17 mL·mg^−1^) exhibited that while this parameter is augmented, the overall rate is generally increased. The findings with POLAG (δ = 0.05; PEG 3000) showed the formation of Form I after 4 min, reaching completion after approximately 30 min. Enhancing δ (0.12 and 0.5) apparently has no direct influence in the cocrystallization efficiency. In POLAG conditions (δ = 0.05; PEG 10,000), the increment in the polymer chain length has no influence in the overall reaction, however, apparently the cocrystallization was slightly slower. At higher amounts of polymer PEG 10,000 (δ = 0.05) at 60 min of reaction, unreacted starting materials were detected.

Interesting results were obtained through qualitative comparison of the rates of cocrystallization by determining the start of the reaction (induction time) and the 50% conversion point performed in NG, LAG, and POLAG experiments. Overall, POLAG and NG showed NG to be almost two times faster. Additionally, in POLAG, the reaction rate was not influenced by the molecular weight of the polymer additives. However, in LAG, the reaction rate was enhanced when η was increased. In addition, the authors indicated that in this reaction, only small quantities of polymer were necessary to have a catalytic role. This work represented an important advance in the influence of the δ parameter in the cocrystal formation as it was mentioned with VALAG.

Another variant of LAG is known as ion liquid-assisted grinding (ILAG), where a salt is added during the grinding as an additive. For instance, the addition of small amounts of salts (NO_3_^−^ or SO_4_^2−^) in the preparation of the MOF [Zn_2_(ta)_2_(dabco)] (ta: terephthalate; dabco: 1,4-diazabicyclo [2.2.2]octane), under ILAG conditions, accelerated the formation of the MOF compared with LAG. [216] The authors suggested that under ILAG conditions, the formation of the MOF [Zn_2_(ta)_2_(dabco)] goes through an anion-templating mechanism.

**Figure 6:**
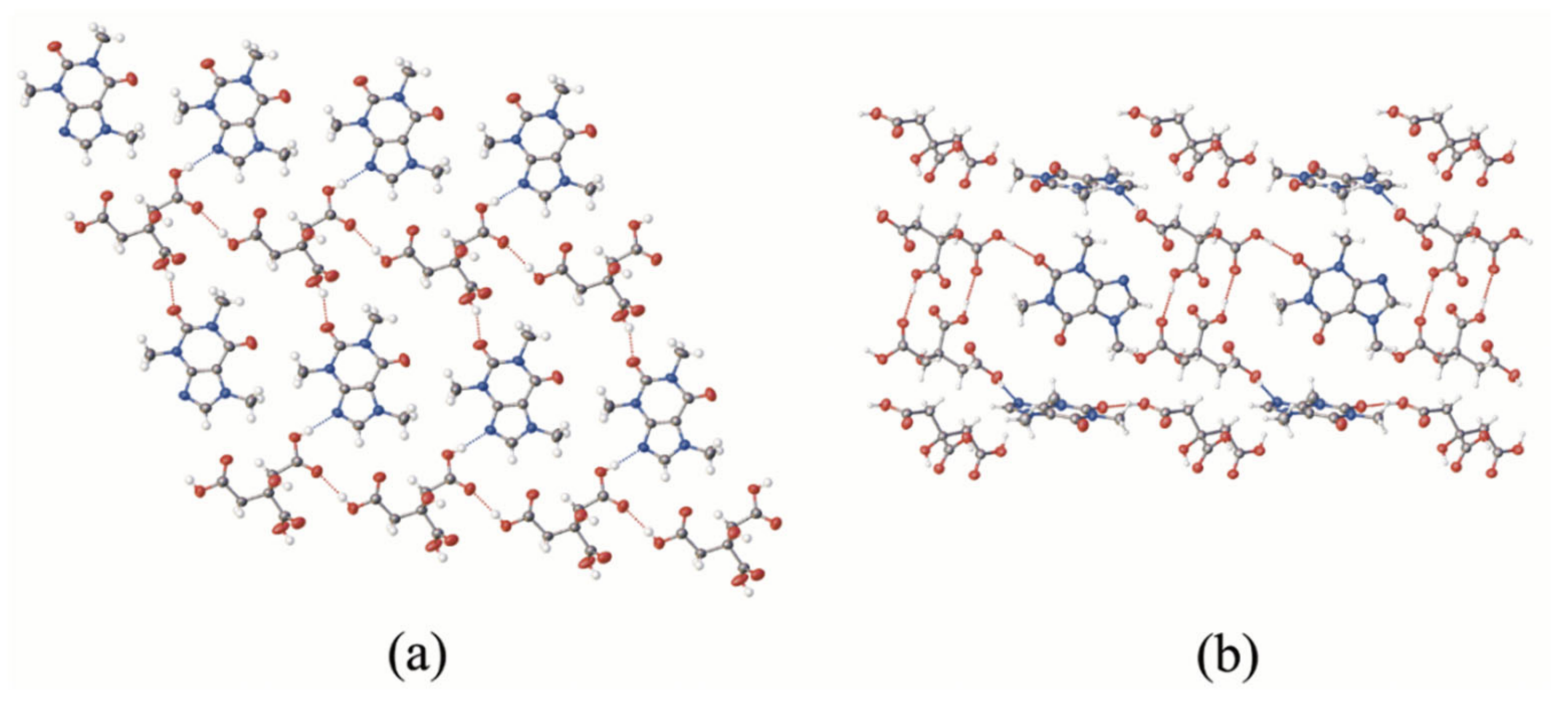
Sheets of caf:ca (1:1) ribbons. (**a**) Form I and (**b**) Form II. CSD refcodes: Form I; KIGKER (Pī) [216] and Form II; KIGKER01 (*P*2_1_/*c*).

**Figure 7:**
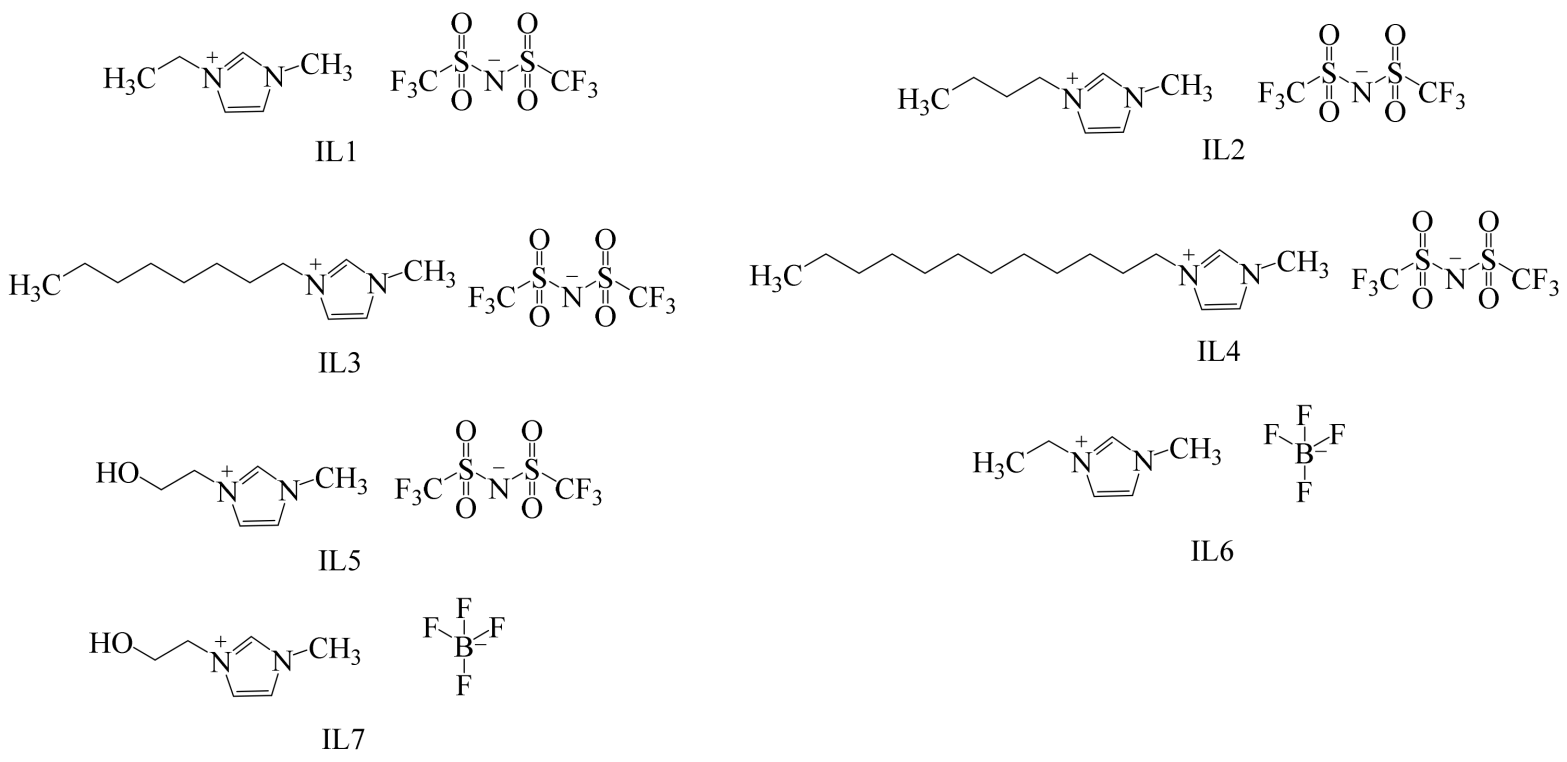
ILs employed in ILAG experiments.

Besides, in 2018, Mukherjee et al., described the formation of the cocrystals caf:ca and caf:glu using ILAG [217]. It must be noted, that for the preparation of the MOF [Zn_2_(ta)_2_(dabco)] by ILAG [216], the grinding was made in the presence of inorganic salts, however, in the formation of the systems caf:ca or caf:glu, different imidazolium-based ionic liquids (IL) were employed. The caf:ca system (1:1) has two cocrystal polymorphs (CSD ref codes: KIGKER (Pī), KIGKER01 (P2_1_/c); Form I and Form II), Figure 6 Form I can be produced by LAG (grinding with water) or NG (only when is used caf hydrated + ca dehydrated) [212]. Form II has only been obtained by slow evaporation in a chloroform/methanol solution [218]. As it was described previously, Form I also can be isolated by POLAG [211]. With this rationale, Mukherjee et al., explored ILAG in an attempt to emulate the results obtained with POLAG, by employing a series of ILs, Figure 7, instead of varying chain lengths of polymers (PEGs). Various ILs were used modifying the substitution on the imidazolium cation (different alkyl chain lengths) or by changing the anions (different hydrogen bonding ability) to determine their influence in the final crystallization outcome.

Experiments in both systems (caf:ca and caf:glu) were carried out in a mortar and pestle in stoichiometric ratio of components (1:1) and adding c.a. 40 μL of the ILs (15 min). The formation of both cocrystal systems was monitored by PXRD.

From the results obtained in the system caf:ca, the authors suggest that independently, the different nature of ILs were employed (hydrophobicity/hydrophilicity), this did not affected the outcome since only the formation of the Form I was observed. However, a different case was observed in the cocrystal system caf:glu. As mentioned above, Form I in caf:glu is obtained by LAG using non-polar solvents. Additionally, Jones et al., reported that the employment of POLAG (increasing the chain lengths of polymers increasing non-polar effect) provides a selective control in the formation of Form I. [219] From this, it has been hypothesized that in both LAG and POLAG, the presence of a non-polar slip plane (200) in Form I absent in Form II, interacts favorably with non-polar liquids. Based on these results, Mukherjee et al., carried out ILAG experiments using hydrophobic and non-polar ILs to stabilize the non-polar slip plane (200) favoring the formation of Form I.

Mukherjee et al., described that employment of all these ILs led to the crystallization of Form I, however the increase in alkyl chain length in the ILs affected the rate of formation. In particular, the use of IL5 promotes the formation of Form I, however, IL7 leads to Form II (polymorph control), by the simple change of the anion, but showed traces of the Form I. With this work, Mukherjee et al., demonstrated that slight modifications in the ILs used in ILAG may promote polymorphic control over cocrystals.

Finally, VATEG (variable temperature grinding) is another subclass of LAG. Here, the monitoring of the temperature changes observed during a mechanochemical process is a relevant factor to reveal reaction kinetics and mechanisms involved [162]. However, VATEG has been scarcely explored and few papers have been reported [220,221]. One of the main hurdles to explore VATEG in ex situ experiments is to monitor the reaction at a given temperature, because it is necessary to interrupt the mechanochemical process to extract a sample for analysis [162]. However, real-time in situ monitoring techniques, in particular variable-temperature synchrotron powder X-ray diffraction, have shown remarkable results to overcome this obstacle. One case study of variable-temperature in situ investigations will be discussed in Section 2.5 [222].

### 2.5. Mechanistic Aspects in Cocrystal Formation Applying Mechanochemistry

Despite the benefits of mechanochemical reactions in the preparation of cocrystals, there is an incipient knowledge behind its formation mechanism. The cocrystal mechanism using mechanochemical methods remains unclear, for this reason, many models have been proposed.

According to Jones and Friščić, the cocrystal formation cannot be assumed as a single mechanism process [117]. At least three different models are accepted to explain these mechanistic aspects: molecular diffusion [117], eutectic formation [223], and mediation by an amorphous phase [224]. These models have a common point, all of them propose the presence of an intermediate bulk phase (gas, liquid, or amorphous form) during the mechanism. Detailed examples explaining these models have been reported [117].

First, under NG conditions using the molecular diffusion model, it is likely to occur when one or both components exhibit considerable vapor pressure in the solid state. In fact, some reactions can proceed simply by contact between the starting chemical reagents, even in the total absence of mechanochemical action. Rastogi et al., reported mechanochemical reactions between picric acid and some aromatic hydrocarbons, where the mechanism is proceeding through surface migration and diffusion via vapor phase [225].

**Figure 8:**
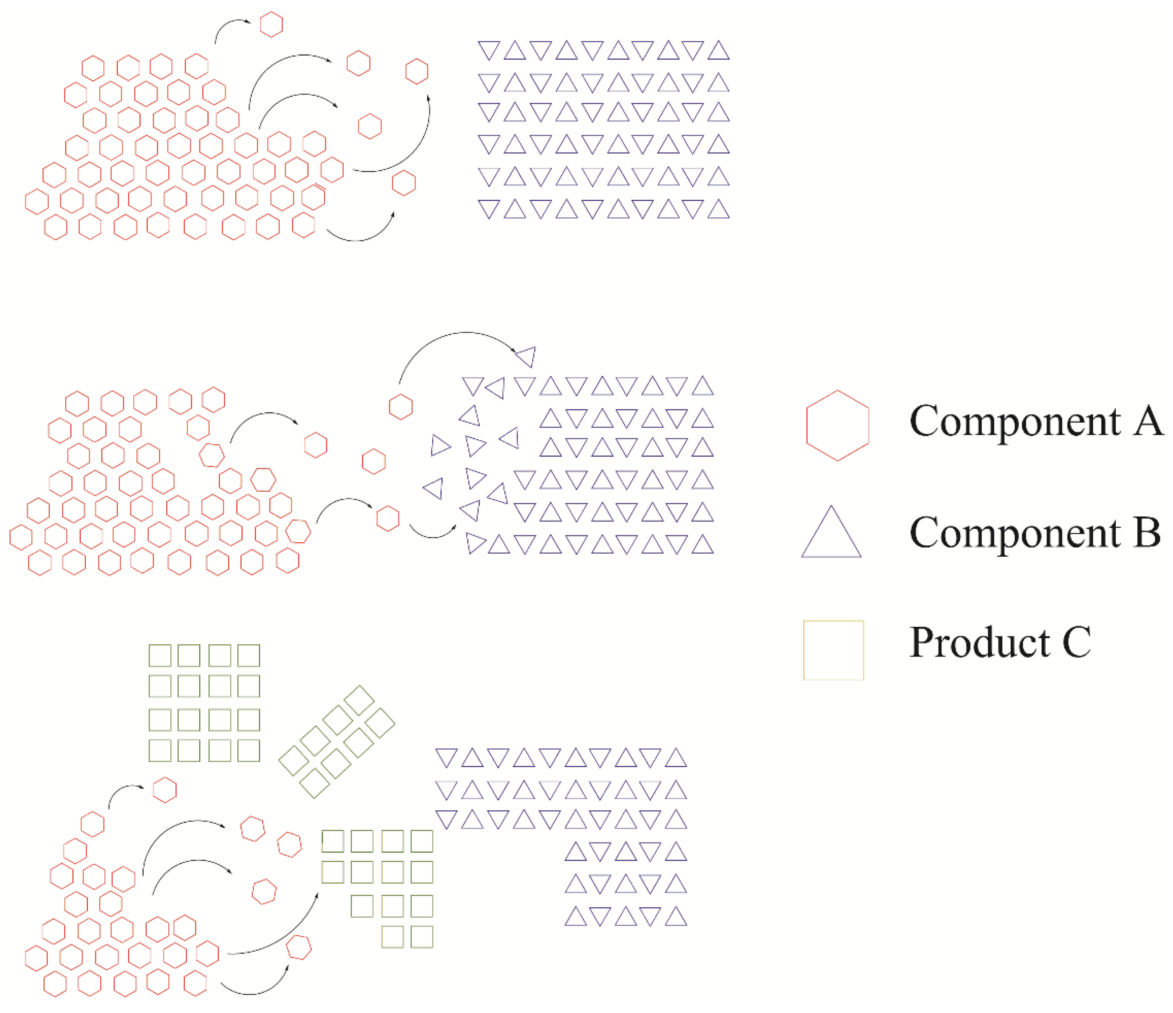
Schematic representation of cocrystal formation mechanism applying grinding/milling in a binary system. Adapted with permission from [117], American Chemical Society, 2009.

In addition, Kaupp contributed with important advances into the mechanistic aspects via molecular diffusion through NG cocrystallization (intersolid reactions), Figure 8 [226]. Using an atomic force microscope, he proposed a three staged mechanism starting from crystal solid species A and B, which give rise to the new product C, Figure 8.

1. Molecule migration. The first stage is the reconstruction of the solid phase, suggesting directional long-range migrations of molecules, where component A invades the planes or channels of component B (or vice-versa). The incipient formation of C distorts the original crystal structures of A and B, producing a mixed A-B-C phase.
2. Product-phase formation. The concomitant appearance of component C in the mixed phase A-B-C favors spatial discontinuity in particles A and B due to strain and crystal defects.
3. Crystal disintegration. In this step, is suggested a chemical and geometrical mismatch between components A and B, produced by the appearance of C causing a disintegration of the particles. The grinding/milling process produces fresh surfaces available for further reaction to completion.

Jones and Friščić suggested that Kaupp’s model although is related for a solid–solid intercrystalline reaction, it may be adopted to explain the formation of cocrystals or multicomponent salts, Figure 8 [117,227].

Additionally, Scott et al., emphasized that some NG reactions between two solids may proceed via an eutectic intermediate (observation of a liquid phase) [228]. According to Chadwick et al., the cocrystal formation (grinding in a mortar) starting from benzophenone and diphenylamine is through an eutectic phase. The authors indicated that the action of the grinding creates a high interfacial area between the starting components facilitating the eutectic form. This induces nucleation and the eventual formation of the cocrystal phase [223].

The cocrystal formation via NG mediated by an amorphous phase is most likely to proceed when the components are non-volatile, especially with solids with strong intermolecular interactions (hydrogen bonds) [117]. Rodríguez-Hornedo et al., demonstrated that grinding carbamazepine and saccharin below the expected glass transition of the final mixture outcome induces an amorphous phase formation. But conversely, when the components are ground at higher temperature of the expected glass transition of the mixture, it would lead to a metastable polymorphic form. The storage of this amorphous form at room temperature slowly tends to cocrystallize. Under high relative humidity conditions (75%), this amorphous phase increases its molecular mobility and complementarity between the components, leading to the cocrystal formation. The amorphous phase intermediate is considered a high energy and high molecular mobility species.

Regarding LAG, the cocrystal formation mechanism is not fully understood [227]. In fact, it has been suggested that the liquid is a lubricant facilitating the molecular diffusion among the components [117,206]. Where the parameter η and polarity of solvent employed plays an important role in the cocrystal formation as it was mentioned with VALAG [207]. Additionally, the parameter η has a strong influence on the possible mechanisms, intermediates, or different polymorphic outcomes in a cocrystallization reaction [229].

It has been suggested that many milling parameters may affect the rates of product formation: milling frequency [203,230], milling time [231], filling degree of the jar [232,233], milling ball diameter and the beaker size [164,234,235], degree of milling ball filling [164], energy input [236,237], and material of jar [231].

Furthermore, variable-temperature in situ studies have shown that reaction rates are temperature-dependent and that slight changes in temperature generally influence the mechanism [222]. Using as a model of synthesis, the preparation of the coordination polymer (CdCl_2_ + cyanoguanidine in dry conditions), the authors showed that an increment of 45 °C in the bulk reaction temperature accelerates the rate reaction (6-fold). The increment in the bulk reaction temperature could be reached by increasing the number of ball impacts, or when are used heavier balls (7 to 9 mm). Raising of the temperature has an important influence in the reactant consumption rate, as well as a change in reaction mechanism. In this context, the authors explain that the accepted model mechanisms for milling reactions in the preparation of inorganic materials (“hot-spot” and “magma-plasma”) [238,239,240], apparently do not properly fit for softer materials such as coordination polymers or cocrystals. According with these results, at temperatures near room temperature, the mechanism goes through an amorphous phase intermediate. However, at higher temperatures, the mechanism proceeds by the rapid formation of an intermediate. Thus, it has been suggested that at elevated temperatures, the mobility of the amorphous intermediate is higher, thus facilitating the formation of the final product via crystallization of the intermediate.

Recently, Friščić et al., demonstrated the appearance of the cocrystal polymorph (Form II) in the system nicotinamide and adipic acid (nic:adi) [229]. It must be noted that the nic: adi cocrystal form has been reported to exist in two stoichiometric relations 1: 1 and 2:1 [241]. Milling nic and adi in a 1:1 stoichiometric proportions using acetonitrile (η = 0.125 μL·mg^−1^) led to the formation of Form I. Having the milling reaction performed using two stainless steel (ss) balls (7 mm) in a poly(methyl methacrylate) (PMMA) material jar.

**Figure 9:**
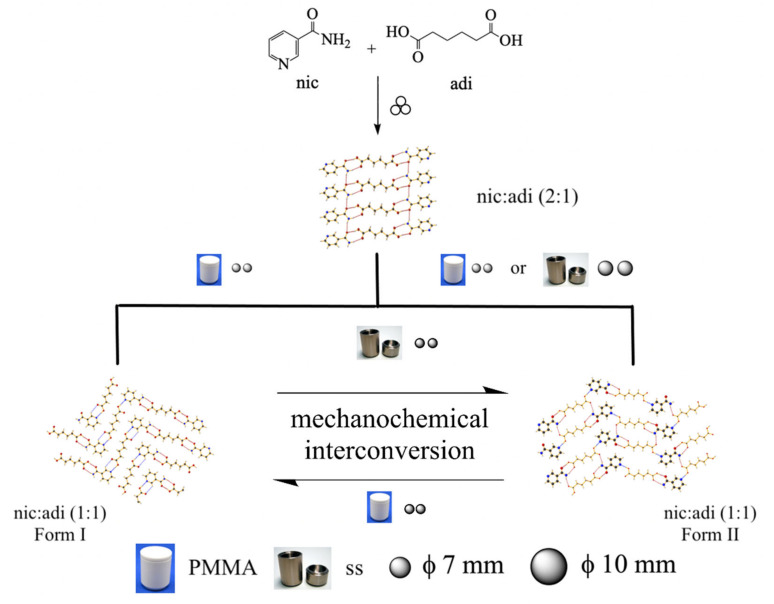
Schematic representation of the different solid forms in the cocrystal system nic:adi. These processes can be controlled by the choice of milling assembly. Adapted with permission from [229], Royal Society of Chemistry, 2020.

Repeating the previous experiment, but using ss jars instead of the PMMA jars lead to the formation of the Form II. The molecular structure of Form II was established by the combination of PXDR and solid state nuclear magnetic resonance (for more details about this technique see Section 3.3) analyses. Besides, in order to verify that the control of the polymorphic outcome depends on the choice of the jar material, experiments under NG and LAG in the presence of different solvents at a fixed η parameter (0.125 μL·mg^−1^) were performed. The solvents chosen were H_2_O, methanol and MeNO_3_. Hence, in LAG reactions, the formation of Form I was preferably observed when PMMA jars were used. Further, Form II tends to be formed when ss jars where employed, Figure 9. In both cases, NG and LAG experiments, the reactions performed with H_2_O were slower, and a mixture of products (Form I and Form II) was observed. These results thus prove that the milling jar material has an important effect in the polymorphic outcome.

The mechanochemical interconversion, Figure 9, can be achieved milling a sample of Form I initially prepared using a ss jar and a pair of ss balls of 7 mm diameter each in the presence of acetonitrile η = 0.125 μL·mg^−1^. From this milling (60 min), Form I can be interconverted to Form II (Form I → Form II). In the case of the reverse process, i.e., Form II → Form I, starting from Form II milling in a PMMA jar under identical conditions, Form I was produced after 2 h, but this reaction was slower and traces of the Form II were detected. It should be noted that the transition enthalpy for the metastable Form II with respect to Form I was determined by differential scanning calorimetry DSC (ΔH = +2.3(3) kJ·mol^−1^). For further details about the DSC technique, consult Section 3.3.

In addition, three different experiments were studied by in situ PXDR. In the first one, (A) PMMA jars and two ss balls of 7 mm diameter were used. Then, in the second and third experiments (B and C), PMMA jars were used, but in experiment B ss balls of 7 mm diameter were added, while in experiment C, two zirconia balls of 10 mm diameter were used. It should be noted that the total balls weight in experiment A, B, and C were 2.8, 5.6, and 5.8 g, respectively.

In the three experiments, the formation of nic:adi (2:1) as the first reaction intermediate was detected, Figure 9. For the experiment A, initially the formation of nic:adi (2:1) is observed and gradually Form I is produced as a final product after 85 min of milling. Further, in experiment B (where balls weight was increased), the reaction proceeded from the intermediate nic:adi (2:1) and the subsequent formation of the Form II and finally produce Form I. The increase in balls weight in experiment B led to an increase in the reaction rate due to a higher energy input. Based on these results, both reaction sequences can be explained under the Ostwald’s rules of stages. These rules describe that: “states that in a crystallization, the system moves to equilibrium from an initial high-energy state through minimal changes in free energy, and thus implying that the least stable polymorph must be the first isolated in any crystallization” [242]. In this context, experiment C showed unexpected results, since the reaction sequence observed was first the formation of nic:adi (2:1) and then Form II (metaestable polymorph) via the thermodynamically more stable phase (Form I). This completely challenges Ostwald’s rules of stages. With this work on mind, Friščić et al., elegantly demonstrated that the polymorphic outcome of the cocrystal system nic:adi is strongly influenced by the nature of the jar material and the balls used.

In another interesting investigation, Guerain et al., demonstrated that cocrystal polymorphism can be observed in the system S or RS-ibuprofen (S-ibp or RS-ibp) with nicotinamide (nic). [243] According to the authors, the polymorphic outcome depends on the synthetic method used. It should be noted that previously both cocrystal S-ibp:nic and RS-ibp:nic were reported by Berry et al. [244] and Guerain et al., synthesized both cocrystals using the following methods: (1) milling under dry conditions, (2) melting at 100 °C and then the mixture was cooled down and then recrystallized (isothermal and non-isothermal processes), and (3) slow evaporation (ethanol). From the results obtained by PXRD or Raman spectroscopy (for further information about this technique, consult Section 3.3), in both cocrystals obtained from methods 1 and 3, the spectral and diffraction information obtained are almost identical. However, the analytical information obtained from method 2 exhibited differences compared with 1 and 3. It is noteworthy that the analytical results observed in the solid form (hereafter Form B) produced by methods 1 and 3 are in agreement with that reported by Berry et al. [244]. This evidence indicated that the solid form obtained from method 2 (Form A) is a cocrystal polymorph of the Form B. Hence, additional PXRD experiments were performed with the products of the room temperature and 100 °C procedures. According to the diffraction results, both cocrystals obtained by methods 1 and 3 remained as Form B until melting. After cooling down from the melting process, both cocrystals were found to be in an amorphous form. The solid form obtained from method 2 corresponds to Form A being slightly different from Form B. This Form A is transformed into Form B at 65 °C, and above 80 °C, the solid form is melted. Further analytical studies by DSC and Raman spectroscopy were performed. The Raman analysis revealed a subtle polymorphic transformation not detected in DCS experiments. Despite DSC techniques being the more suitable technique to analyze phase transitions, non-detection of the polymorphic transformation was observed, suggesting that both Forms are slightly different from an energetically point of view. Since the polymorphic transformation was not detected by DSC, analysis by low-wave number Raman experiments under similar thermal conditions employing the same calorimetric technique (DSC) led to the observation of two-step successive mechanism. From recrystallization experiments (method 2, isothermal and non-isothermal), it was first observed the formation of Form A via a nano/microcrystalline state. Being the second step, the transformation of Form A into Form B. It was observed that transformation of Form A into Form B upon heating via the two-step mechanism exhibited very weak changes in the hydrogen bond networks of the participant intermediates. This work thus demonstrated the crucial role of the H-bonding organizations on the cocrystal formation mechanisms.

**Figure 10:**
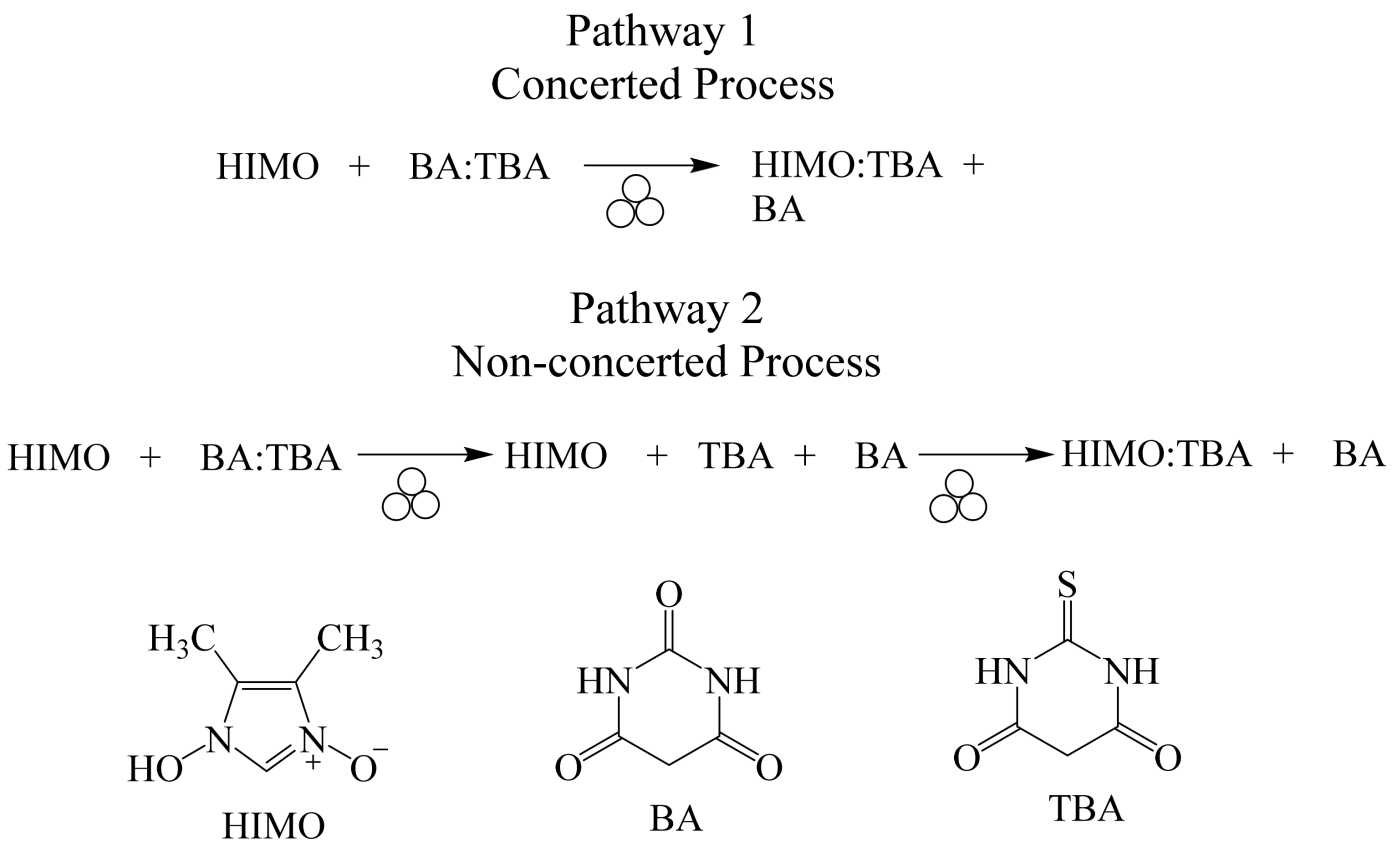
Schematic representation of the two proposed mechanisms behind the formation of HIMO:TBA.

Finally, in another interesting study, Dudek et al., prepared, by ball-milling using methanol as an additive, a ternary cocrystal system starting from a binary cocrystal system. Evaluation of the possible pathways involved suggests a non-concerted mechanism [245]. The components involved were barbituric acid (BA), thiobarbituric acid (TBA), and 1-hydroxy-4,5-dimethyl-imidazole 3-oxide (HIMO), Figure 10. Using the binary cocrystal system BA:TBA; 1:1 as model, [246] and comilled with HIMO, the reaction was monitored by ssNMR (for further information about this technique, consult Section 3.3), Figure 10. Based on the results obtained, two mechanistic pathways were assumed. Pathway 1 involving a concerted process, in which, as HIMO is forming the new solid form (HIMO:TBA), BA is simultaneously departed in a synchronized manner. Additionally, the non-concerted Pathway 2, where it is proposed that in the intermediate stage the three components are completely separated and subsequently HIMO:TBA is formed.

According to the ssNMR experiments and theoretical calculations, this mechanochemical reaction leading to the formation of HIMO:TBA follows a non-concerted mechanism. It is important to remark that authors indicate that both pathways are equivalent to the mechanisms observed in organic chemistry (S_N_1 and S_N_2 reactions).

## 3. Cocrystal

### 3.1. Cocrystal Definition

For many years, there has been an intense debate in the attempt to propose a formal definition of what a cocrystal is [18]. Thus, several variations and modifications in the definition have been made in the last years [48]. The difficulty to have a proper definition of a cocrystal is due to the diversity observed in the scale range-order periodicity and composition amongst the different multicomponent solid forms of an API, Figure 1 [15,247]. Apparently, before the contributions of Aitipamula et al. [18] and Groethe [56], it was somewhat difficult to delimitate the cut-off amongst polymorphs, solvates, hydrates, molecular salts, polymorphs of cocrystals, etc. However, it seems that Aitipamula and Groethe’s approaches have had a great impact in the development of a simplistic way to classify and differentiate all of this.

**Figure 11:**
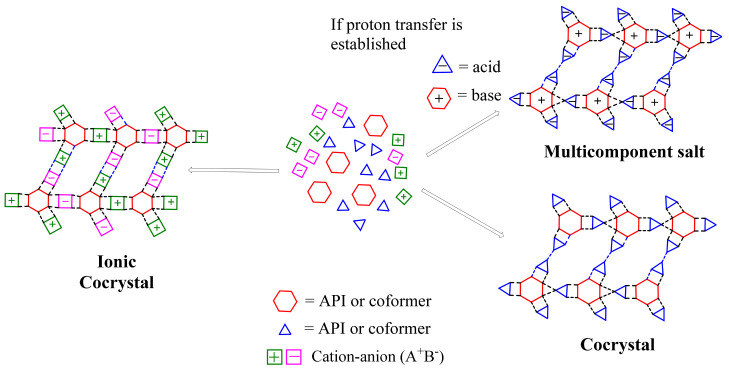
Schematic representation of the formation of multicomponent salt or cocrystal or ionic cocrystal.

By itself, the term cocrystal is referred as a crystalline molecular complex, in which the components are neutral species forming an adduct, otherwise it should be considered a multicomponent salt (charged anions and cations) [248]. Thus, in a binary system (A: acid; B: base), a salt is formed when a proton transfer exists establishing the molecular specie (A^−^ B^+^-H) [60]. Thus, from the Crystal Engineering context, the formation of multicomponent salts comes from the combination of acids and bases, and cocrystals are not necessarily made up from these species, Figure 11 [248]. Thus, it is noteworthy to mention three important considerations described by Aakeröy et al., to define a cocrystal [249].

1. “Only compounds constructed from discrete neutral molecular species will be considered cocrystals. Consequently, all solids containing ions, including complex transition-metal ions, are excluded”.
2. “Only cocrystals made from reactants that are solids at ambient conditions will be included. Therefore, all hydrates and other solvates are excluded which, in principle, eliminates compounds that are typically classified as clathrates or inclusion compounds (where the guest is a solvent or a gas molecule)”.
3. “A cocrystal is a structurally homogeneous crystalline material that contains two or more neutral building blocks that are present in definite stoichiometric amounts”.

In addition, some authors have mentioned that the prediction of whether a cocrystal or salt will be formed can be on basis of the ΔpKa rule [250,251]. This empirical consideration refers that a salt is expected to be formed when a ΔpKa value between the acid and the base is greater than 3 (ΔpKa > 3), thus observing a proton transfer (A^−^:B^+^-H) [13,61,252]. Further, when the value is ΔpKa < 0, the product is a neutral cocrystal (proton is retained on the acid). However, many cocrystallization attempts may fall in the range of 0 < ΔpKa < 3, which will form mixed ionization states between components (proton is in-between the acid and the base) [253]. These multicomponent forms found in this situation are denominated salt-cocrystal continuum [48,61,254].

**Figure 12:**
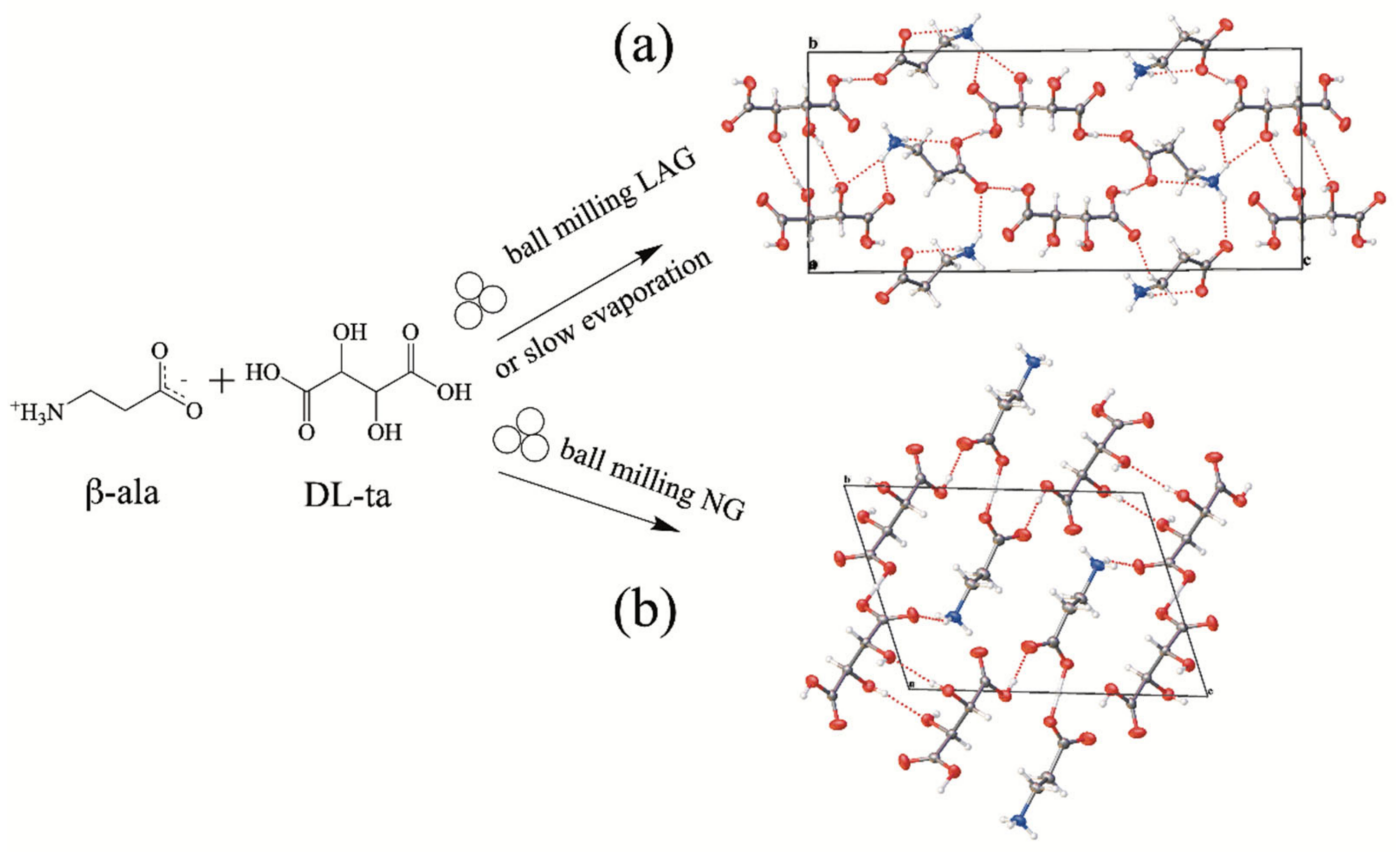
Formation of different multicomponent solid forms (β-ala + DL-ta; 1:1) depending on the synthetic method used: (**a**) cocrystal (plane *bc*; CSD refcode: VELBIA) and (**b**) multicomponent salt (plane *bc*; CSD refcode: VELBIA01).

From these borderline concepts, apparently, the distinction between cocrystal vs. multicomponent salt is well defined. However, difficulties have emerged when the ionization state of the charged species can change upon variations in temperature [255]. Additionally, few papers have reported binary systems where some multicomponent pharmaceutical solid forms crystallize concomitantly as cocrystals and salts in the same stoichiometric proportions [256,257]. In fact, recently, Boldyreva et al., reported that the formation of a cocrystal or a salt is dependent of the synthetic method used [258]. Boldyreva et al., specifically mentioned that the binary system β-alanine (β-ala) and DL-tartaric acid (DL-ta) in 1:1 stoichiometry is an interesting case of study since by ball-milling under liquid assisted conditions and/or by slow evaporation crystallization, the cocrystal form can be produced (CSD refcode: VELBIA; P2_1_/c). However, by ball-milling under dry conditions, the multicomponent salt is formed (CSD refcode: VELBIA01; Pī), Figure 12.

Aitipamula et al., indicate that the FDA definition of a cocrystal is ambiguous: “solids that are crystalline materials composed of two or more molecules in the same crystal lattice” [18]. Therefore, they proposed a more ample and broader definition: “cocrystals are solids that are crystalline single-phase materials composed of two or more different molecular and/or ionic compounds generally in a stoichiometric ratio” [18]. Recently, Zhang et al., redefined the term cocrystal in an attempt to achieve a more uniform definition: “a cocrystal is a single-phase crystalline solid composed of two or multiple components in a stoichiometric ratio, and the components of a cocrystal can be atoms, molecules, anions, and cations in pairs, and/or metallic cations with free electrons shared [259].” According to this definition, cocrystals can be subdivided in five groups depending of the type of the components and their interactions in the crystal entity: atomic cocrystal, molecular cocrystal, ionic cocrystal, metallic cocrystal, and mixed-type cocrystal [259]. It should be noted that typically, ionic cocrystals are defined as multicomponent materials composed from a salt (or an ionic compound) and a neutral organic molecule [57,58,260]. Ionic cocrystals are defined in general as A^+^B^−^N where A^+^ is a cation, B^−^ is an anion, and N is a neutral molecule, Figure 11.

Thus, a straightforward form to view a pharmaceutical cocrystal is *a crystalline single phase where in the crystal lattice of an API is incorporated another neutral component denominated coformer (cocrystallizer agent or cocrystal former) in a specific stoichiometric ratio* [55,58]. In this context, the incorporation of a coformer alters the physicochemical and biological properties of the parental API (e.g., solubility, thermal stability, dissolution rate, bioavailability) without any covalent modification [57]. Both API/coformer are held together by non-covalent interactions (van der Waals contact forces, π-π interactions, and/or hydrogen bond). The coformer to be incorporated in the crystal lattice of an API must be selected as a pharmaceutical molecule accepted, Generally Recognized as Safe (GRAS) [57,261]. On the other hand, agglutination of two or more APIs into one unit dose yields the formation of a drug–drug cocrystal also known as multidrug cocrystal [227]. Recently, the preparation of drug–drug cocrystals has gained a lot of interest in combination therapy [57,262]. The importance of these multidrug materials lies mainly in their potential activity in the treatment of complex diseases which require the simultaneously administration of two drugs [64,263,264]. Despite the potential therapeutic properties that may show drug–drug cocrystals, few examples are found marketed (Entresto™: Valsartan sodium–sacubitril sodium) and some are encountered in clinical trials (Esteve™: tramadol hydrochloride–celecoxib) [20,265].

Another important point to highlight is that from the legal and regulatory perspective, pharmaceutical cocrystals are a patentable product [266,267].

### 3.2. Design of Pharmaceutical Cocrystals: Selection of Appropriate API/Conformer

The ability of an API to cocrystallize in the presence of a coformer depends on diverse parameters: stoichiometry ratio (API/coformer), solvent(s) employed, temperature, and crystallization technique used. Usually an API is more apt to cocrystallize when possessing donor and acceptor sites able to form reliable hydrogen bonding (supramolecular synthons [268]) with the coformer [14,269]. The selection of an appropriate coformer is mainly on basis of hydrogen bond propensities, molecular recognition, and innocuousness (GRAS), nevertheless these considerations do not guarantee the formation of the cocrystal [12,270]. For the design of pharmaceutical cocrystals, there are two approaches: (1) identification of complementary sites of the API and the coformer to form reliable hydrogen bonds, and (2) the preparation of cocrystals based on high throughput screening crystallization [5,263]. The first approach is mainly based on the precepts of the graph set theory (Etter’s rules) [271]. The second approach may result time-consuming and expensive. In addition, the help of computational and informatics approaches are recurrently used as tools in the prediction and selection of suitable API/coformer candidates [272,273,274]. Recently, Aakeröy et al., reported a systematic process to find a proper selection of coformers based on hydrogen-bond propensities using the CSD database [275].

### 3.3. Characterization of Pharmaceutical Cocrystals

Single crystal X-ray diffraction (SCXRD) is by far the most preferable technique to gain information on molecular recognition, hydrogen bonding patterns, assembly and packing of pharmaceutical cocrystals [276,277]. SCXRD is an excellent technique to make distinction between the formation of cocrystal vs. multicomponent salts [278,279], and salt-cocrystal continuum forms [61]. Unfortunately, in most of the cases obtaining suitable single crystals for X-ray diffraction is not easy, and other analytical methods must be used to gain knowledge about the formation of cocrystals.

Among these other techniques for the characterization of cocrystals, we found PXRD. This diffraction analysis technique is extensively used for the characterization of different multicomponent solid forms: cocrystals/salts [276,277], eutectics solid mixtures [63], coamorphous [52], allowing to understand the structural properties of these materials. In some cases, simulated molecular structures can be predicted from PXRD data [14]. The employment of software programs as DIFFRAC.TOPAS (Bruker AXS, Karlsruhe Germany) [280] or DASH [281] help in the structural determination of molecular structures based on Rietveld analysis.

Thermal analysis methods are of high importance in the structural characterization of cocrystals; for instance, DSC, differential thermal analysis (DTA), and thermogravimetric analysis (TGA) [14,276,277]. These analytical methods help to determine the formation of new solid phases; to determine percentages of crystallinity and percentages of weight loss, providing quantitative measurement of the associated enthalpy change, observation of thermal transitions, and detection of polymorphism, etc. DSC can prove the formation of cocrystals, since the melting point determined is usually observed between the melting temperatures of the pure components. However, some DSC cocrystal thermograms exhibit consecutive multiple peaks. When DSC thermograms show two peaks, the first is due to the eutectic melting and the second corresponds to the cocrystal melting. In some cases, DSC scans may show three peaks, the first again corresponding to the eutectic melting, the second endotherm indicates the melting of the excess of one of the components (API or conformer) and the third corresponds to the cocrystal melting. Additionally, the appearance of multiple peaks in DSC scans could be associated with a polymorphic transformation of cocrystals [48].

**Figure 13:**
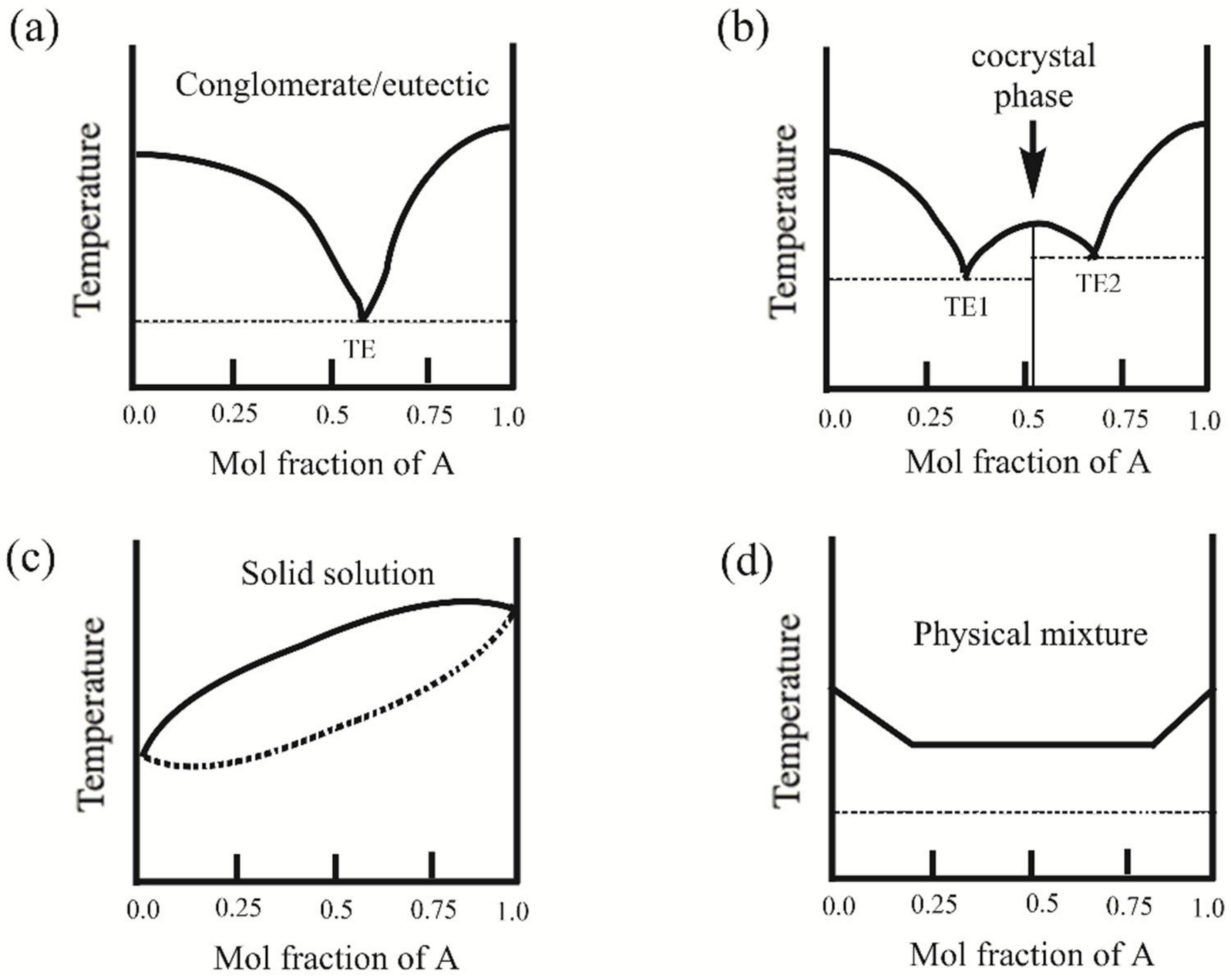
Schematic representation of binary phase diagrams (components A + B). (**a**) Solid eutectic mixture; TE: eutectic temperature. (**b**) Cocrystal formation; TE1 and TE2: eutectic temperatures 1 and 2. (**c**) Solid solution formation. (**d**) Physical mixture. Adapted with permission from [48], MDPI, 2018.

Currently, the construction of binary phase diagrams (components A + B) based on DSC data is fundamental to distinguish among different solid forms: eutectic mixtures, cocrystals, solid solutions, physical mixtures, etc., Figure 13 [13,48].

The construction of binary phase diagrams are made from data obtained from DSC scans at different compositions [282]. The formation of a binary eutectic mixture can be proved by the observation of the characteristic V-shaped diagram (depression in the melting point, TE: eutectic temperature, Figure 13a).

On the other hand, a pharmaceutical cocrystal has a W-shaped graph (Figure 13b). A W-shaped diagram exhibits at least two or more eutectic points (TE1 and TE2) and among them is found the cocrystal phase, Figure 13b [283]. Thus, the screening and detection of pharmaceutical cocrystals [284,285], solid eutectic mixtures [286,287], solid solutions [288], and physical mixtures (in binary systems two endotherms are observed in DSC thermograms corresponding of each component) [289] can be carried out by DSC analysis.

Additionally, ternary phase diagrams (component A + component B + solvent) help in the determination of the suitable stoichiometric ratio for the formation of a cocrystal. These types of diagrams are employed when cocrystals are not formed in a particular solvent due to the fact that both components exhibit incongruent solubility during solution crystallization [48,117].

Spectroscopic analysis is also widely used in the cocrystal characterization. According with Pindelska et al., there are two types of spectroscopic characterization methods: vibrational and nuclear magnetic resonance (NMR) [276]. Vibrational methods span from Fourier transform infrared spectroscopy (FT-IR), Raman scattering and Terahertz spectroscopy.

Specifically, a FT-IR spectrum shows the frequency, shape, position, and intensity of a peak related with the absorption of a vibrational mode associated with a functional group. Raman spectroscopy on the other hand, exhibits vibrational and rotational modes at low frequencies, mainly nonpolar functional groups that FT-IR cannot detect. Terahertz time-domain spectroscopy on the other hand, records frequencies in the interval (0.1–10 THz, which is 3.3–333.6 cm^−1^), where bands correspond to vibrations associated with intermolecular interactions (hydrogen bonds, van der Waals, etc.) [276].

Another spectroscopic technique gaining relevance lately is X-ray photoelectron spectroscopy (XPS). This technique is capable of exhibiting the changes involved in the chemical environment of a substance as a result of the supply of different amounts of energy (binding energy), required to emit an electron from the nucleus at the atomic level. Fundamentally, XPS records the binding energy (kinetic energy) of photoelectrons emitted from a sample after being irradiated with X-rays [290,291]. XPS can distinguish between the formation of a cocrystal or a multicomponent salt. However, although Tothadi et al., indicated that the reliability of XPS in the distinction between a cocrystal and a multicomponent salt is robust, the determination of a salt-cocrystal continuum is difficult to distinguish [253].

Solid-state NMR (ssNMR) is another important technique to gain more valuable insights in the cocrystal characterization [248,276,277]. Recently, a new approach denominated NMR crystallography has been developed [276]. This approach combines the long-order data provided by PXRD and short-order data obtained by ssNMR (e.g., local symmetry, number of molecules contained in the unit cell, orientations, and non-covalent distances of the molecules). NMR crystallography provides a complete characterization using molecular modeling and quantum chemical to offer a prediction of the crystal structure when structures cannot be determined by SCXRD [292].

### 3.4. Diverse Preparation Methods of Pharmaceutical Cocrystals

The preparation of cocrystals comprises diverse strategies: refs. [48,227,293] dissolutive cocrystallization methods, antisolvent addition, slurry conversion, sublimation, vapor digestion, hot-stage microscopy melt interface, supercritical fluids, sonic slurrying, melt extrusion, wet compression, dry compression, microwave synthesis, and various solid-state synthetic methods, including NG, LAG, POLAG [293], TSE [48,182,183,294,295], resonant acoustic mixing (RAM) [296,297,298,299], ultrasound methods, etc.

TSE offers a unique opportunity not found in grinding or milling approaches because they are difficult to scale up to manufacture large amounts of the desired cocrystal [298]. In fact, one of the benefits of TSE is that the process of crystallization can be carried out under solventless conditions [181]. Alvarez-Núñez et al., demonstrated that TSE can be an important method of preparation of cocrystals at the large scale by studying four different cocrystal systems [182]. The authors emphasize that temperature and extent of mixing are the main parameters for a successful conversion in the process. TSE equipment consists basically of two co-/counter-rotating screws in a single barrel. By mechanical action of screwing, the components are mixed along the length of the barrel forming cocrystals [227].

RAM is another technique that deserves to be described in this review, since within the branches of mechanochemistry, sonochemistry considers chemical transformations produced by the action of sound, Figure 3 [80]. RAM was born as an alternative to mechanochemical synthesis, because the considerable amount of mechanical energy transferred from milling bodies to the sample may cause damage to particles, or large numbers of defects into the crystalline lattice or complete amorphization. Mainly, RAM is an alternative for the preparation of highly sensitive-explosive materials and propellants. Furthermore, RAM is recommended when the desired outcome is required to have high crystallinity. Besides, another benefit found in the use of RAM is the scale-up possibility for the formation of a given cocrystal [298,299].

Basically, RAM is a technique where the components are mixed intimately at high frequency, promoting the formation of cocrystals. The acoustic energy is generated by the action of the oscillation of springs, which induce mechanical resonance, frequency directly transferred to the sample vessel originating local mixing zones [296].

An interesting case of the scale-up benefits of RAM in cocrystal production is reported by Nagapudi et al. [299]. The authors carried out high throughput screening to 16 cocrystal systems previously reported. A 96-well plate was adapted to screen the 16 cocrystal systems using RAM. The screening experiments at each well were carried out at different conditions: (i) components with no zirconia beads, (ii) components with water, (iii) components with zirconia beads and water, (iv) components with ethanol, (v) components with zirconia beads and ethanol.

According with the number of hits obtained after the screening of the 16 cocrystal systems, the best conditions found were when solvent was added (13 out of 16). Further, the worst conditions (3 out of 16) were when no solvent or beads were added. Under conditions of adding beads without solvent, moderate hits were obtained (5 out of 16). Then, Nagapudi et al., reported that solvents played a crucial role in the screening. The use of beads and solvent showed similar results as those observed in the hits with just the use of solvents. The liquid assisted high-throughput screening using RAM is an effective technique to produce cocrystals. In addition, the cases where the screening were unsuccessful could be due two factors: (i) wrong choice of solvent or (ii) no proper selection of energy-input.

In a second experiment, the theophylline-citric acid (thp:ca) system was used as a model. The cocrystal system thp:ca can be prepared by NG or LAG [212]. The cocrystallization outcome is dependent of the conditions used. If one of the initial components is hydrated or water is added during the grinding, the cocrystal hydrated form is obtained. Otherwise, if both components are anhydrous or the solvent used is not water, then the anhydrous cocrystal form is yielded. In this case, Nagapudi et al., reproduced all the above-mentioned experiments using RAM. The experiments were carried out with 50 μL of either ethanol or water. In addition, essays were conducted with and without beads. From these results, it was observed that both forms can be obtained by RAM (anhydrous and hydrated). Experiments using simultaneously beads and solvent exhibited complete conversion of the reaction. However, essays only employing solvent, presented unreacted amounts of thp. It was observed that addition of beads accelerates the kinetics of the conversion.

Another set of experiments were carried out evaluating η at different values (0.0, 0.25, 0.5, 1.0, and 2.0, using water). The cocrystal system used was thp and oxalic acid (ox) in a 2:1 stoichiometry. The results obtained from η = 0.0 to 1.0 showed that the reactions almost completely led to the formation of the cocrystal thp:ox. However, when η = 2.0 exhibited unreacted thp. It should be noted that solubility in water of thp is ~8.3 mg/mL whilst ox is ~143 mg/mL. Under η = 2.0, probably ox is completely solubilized and thp not. It is important to note that at η = 2.0, a significant conversion to the cocrystal thp:ox is observed, but due to the lower solubility of thp compared to ox, this hampers the completeness of reaction.

Besides, scale-up experiments were carried out using the cocrystal system thp:ox. For the scale up entry at 80 g, it was observed that the successful formation of the cocrystal depends at least on two parameters, acceleration and mixing time. For instance, the first 30 min of mixing at 60 g the conversion to the cocrystal reaches 97–98%. No perceptible changes in conversion were detected increasing from 30 min to 6 h. In addition, increasing to 10 h of mixing led to a similar conversion percentage as that observed at 30 min. However, according to PXRD data, it is possible that the thp experiences polymorphism. According to these results, an excellent balance between acceleration and mixing time is important for the formation of the cocrystal.

In general, Nagapudi et al., indicated that TSE and RAM are important techniques for the scaling up of cocrystals. TSE seems not to be effective for the cocrystal screening but provides higher shear and access to a greater temperature range than RAM. When cocrystal formation occurred by an eutectic intermediate, TSE is more appropriate than RAM. In scale up proportions, RAM is suitable to produce hundreds of grams to 1 kg of the desired cocrystal while TSE is appropriate for larger manufacturing scales (various kilograms).

**Figure 14:**
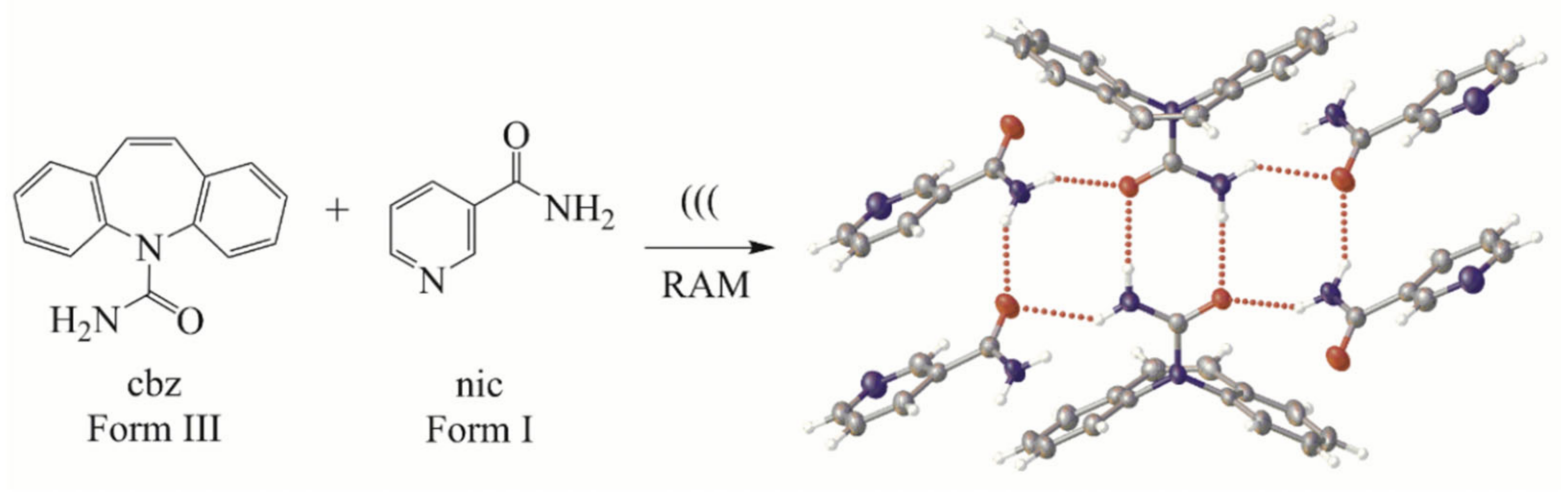
Cocrystal formation of cbz:nic 1:1 (CSD refcode: UNEZES; P2_1_/n) using RAM.

Michalchuk et al., reported the first in situ real-time study of cocrystal formation by RAM using synchrotron X-ray radiation and a Resodyn LabRAM instrument [296]. The cocrystal system used as the model was carbamazepine:nicotinamide (cbz:nic 1:1; CSD refcode: UNEZES; P2_1_/n) [300] starting from cbz Form III + nic Form I, Figure 14. Previously, it has been reported the cocrystallization of cbz:nic 1:1 using RAM [298].

RAM experiments under dry conditions did not produce the cocrystallization product between cbz and nic. Nevertheless, the reaction proceeded after addition of one drop of water (ca. 20 μL). The experimental conditions of the Resodyn LabRAM instrument were kept fixed with respect to the magnetic resonance frequency approximately 61 Hz, however, the amplitude of the oscillation was adjusted to two acceleration values 50 and 100 G.

After 90 s of mixing of both components under 50 G using RAM, Rietveld refinement of the in situ PXRD data indicated that the major product detected was the dihydrated form of cbz (cbz·2H_2_O). The formation of cbz·2H_2_O implies that this is the kinetically controlled product. Additionally, according with the Rietveld refinement, after 90 s of reaction, only 10 mol% of the cocrystal cbz:nic 1:1 is formed.

Substantial acceleration in the cocrystal formation of cbz:nic 1:1 was observed under 100 G. During the first 30 s of mixing, it was detected only traces of the initial components and the cocrystallization occurred rapidly. Contrary to the Rietveld refinement observed at 50 G, suggesting the formation of the product cbz·2H_2_O, at 100 G, the dominant phase was the cocrystal cbz:nic 1:1.

Therefore, the authors suggested that higher accelerations conditions led to a more rapid mixing of the components. Higher accelerations promote more reaction zones (comminution, particle–particle interfaces, surface regeneration, etc.) favoring the intimate mixing, while at lower accelerations particles tend to be agglomerated inhibiting this process. According to the authors, temperatures reached in situ in the sample jar at different acceleration conditions vary slightly (ca. 40 °C).

Cocrystal formation by sonochemistry is another technique that is worth mentioning [124]. Sonochemistry is based on the formation of acoustic cavitation (formation, growth, and implosive collapse of bubbles in liquids) caused by the action of mechanical effects of sound. Application of ultrasound generates interruption and breaking of attractive forces between the molecules of the liquid medium, causing a considerable drop in the internal pressure forming bubbles. Bubble collapse or collision creates unique conditions: temperatures above 5000 K, pressures exceeding 1000 atmospheres, and heating and cooling rates in excess of 10^10^ K·s^−1^ providing mechanical energy [80].

Some examples using sonochemistry for the preparation of cocrystals have been reported [206,301]. It should be noted that sonochemistry processes typically proceed, adding a drop of solvent or under slurry conditions. Usually, all the papers reporting sonochemical reactions mentioned were conducted under ultrasonic cavitation of a liquid phase (formation of microbubles). However, recently, Roy et al., published that preparation of cocrystals by this method can be performed under dry conditions employing an ultrasonic cleaning bath [124]. Specifically, the report of the formation of two cocrystal systems; first, paracetamol in the presence of caf; 4,4′-bipy or 5-nitroisophthalic acid (5-nip) each in a 1:1 ratio. Additionally, the other cocrystalline form is meloxicam:aspirin (1:1) reported previously by Zaworotko et al. [302].

Initially, for the systems using paracetamol, each pair of solids were sonicated for 60 min in a glass vial sealed and suspended in an ultrasonic cleaning bath under dry conditions. Analysis by PXRD of the solid forms paracetamol:caf and paracetamol:4,4′-bipy exhibited the successful formation of the cocrystals. However, the system paracetamol:5-nip only showed the unreacted starting components by PXRD. Another set of time-dependent experiments were carried out to determine how the percentage of conversion towards the cocrystal was affected. Samples of paracetamol:caf and paracetamol:4,4′-bipy were sonicated for 15, 30, 45, and 60 min. The full conversion for the cocrystalline product in both systems was observed only at 60 min. For the cocrystal formation of paracetamol:5-nip, it was necessary to micronize the powders in particle size of <500 or <150 μm. Decreasing particle size enhances the rate of reaction since the surface area was increased. Sonication experiments using micronized powders were unsuccessful in the transformation to the cocrystal phase. Thus, liquid assisted sonochemical irradiation (LASI, analogous to LAG) was applied. The successful conversion into the cocrystal form paracetamol:5-nip was observed, sonicating the powders by 60 min using methanol (1.0 mol·eq). Additionally, experiments at different times of sonication (15, 30, and 45 min) and employing different LASI conditions (0.25, 0.5, and 0.75 mol·eq) were carried out. The reaction at 15 min and in the presence of 0.25 mol·eq of methanol was quantitative. Therefore, the authors concluded that adding solvent in catalytic amounts dramatically accelerates the reaction. Additionally, they suggested that despite the melting points of the starting materials being high, formation of a melt as an intermediate during the reaction is not discarded.

Regarding the cocrystal system meloxicam:aspirin, produced by sonication of both components in complete dry conditions, analysis by PXRD exhibited the formation of the desired solid form. However, traces of the starting constituents were observed. Additionally, the starting powders were micronized and sonicated by 60 min. The resulting PXRD patterns indicated that the reaction occurred, however the crystallinity of the product was very low. With these results, it could not be categorically concluded the formation of the desired cocrystal. Thus, in an attempt to optimize the reaction LASI was carried out using CHCl_3_ (10.0 mol·eq) sonicating by 60 min. Being these conditions enough for the complete formation of the desired cocrystal. Thus, globally, this work demonstrated that ultrasonic cavitation of the liquid phase (formation of microbubbles) is not necessary, since some reactions were carried out in the absence of solvent.

### 3.5. Benefits of Pharmaceutical Cocrystals

Cocrystallization methods have emerged as a Crystal Engineering approach in an attempt to modify the solid-state properties of an API without affecting its intrinsic properties [303]. The implications of these solid-state modifications by cocrystallization procedures rely mainly in the improvement of properties as: solubility [47,48,49,304], dissolution rate [45,46], bioavailability [55,304,305], permeability [304], tablet ability [306,307], and thermal stability [308], or taste masking [55].

### 3.6. Chronological Survey of Papers Mentioning Mechanochemical Synthesis of Pharmaceutical Cocrystals

Various scientific reports are available in the literature describing the formation of diverse pharmaceutical cocrystals by mechanochemical methods. These mechanochemical methods span from grinding/milling under dry or liquid assisted conditions, TSE, RAM, impact/shear/vibratory treatment, POLAG, ILAG, etc. In this regard, Table 1 shows a chronological summary of the most representative publications associated with these procedures. Table 1 is related with the formation of pharmaceutical cocrystal or multicomponent salts (and all their different solid forms described in Figure 1 and Section 3.1) of the type API + coformer or API + API forms (multidrug). In this survey, also were included natural products (NP hereafter) instead an API. NP should exhibit pharmaceutical activity. The survey was made using Scifinder^®^ (Columbus, OH, USA) under the restriction search “Liquid assisted grinding cocrystals or neat grinding cocrystals or milling cocrystals or grinding cocrystals or grinding salt-cocrystal continuum”. The survey was made for the period of 16 November 2020 to 5 February 2021. Patents were not included in this survey.

## 4. Conclusions

In conclusion, we have highlighted most of the benefits found in the field of the mechanochemistry particularly in the formation of pharmaceutical cocrystals. Mechanochemistry offers unique opportunities in a green approach, eliminating or minimizing almost entirely the use of solvents. It must be noted, that mechanochemistry has an extensive set of advantages compared with classical solution synthesis. For instance, the selective formation of solid or polymorphic forms that would not otherwise be obtained by solution-based synthesis. Additionally, the decrease in energy consumption, reduction in the generation of residual solvents, etcetera.

Despite the benefits mentioned above, the emerging understanding of mechanistic factors leading to the formation of pharmaceutical cocrystals through mechanochemistry, has limited the use of these methods, because of the significant differences often found between reaction outcomes compared to classical solution methods. This must trigger further studies of these procedures with the aim of prioritizing these ecofriendly reactions. Besides, this review makes a chronological recount of the most relevant publications dealing with relevant studies related to mechanochemical synthesis of pharmaceutical cocrystals.

In this sense, mechanochemistry can be seen as an attractive prospective green tool, not only for the preparation of cocrystals, but also finding many applications in other fields (e.g., physics, chemistry, materials science).

## Figures and Tables

**Table 1:** Chronological summary of the most representative publications related with the formation of pharmaceutical cocrystals or multicomponent salts by mechanochemical procedures.

API or NP + Coformer or API + API	Year of the Paper	Type of Grinding or Method of Synthesis and Characterization	Type of Multicomponent Form	Reference
Adenine + Thymine	1993	NG/slow evaporation	Cocrystal	[309]
Sulfadimidine + 8 diverse Carboxylic acids coformers	1995	NG	Cocrystal	[310]
Caffeine + Glutaric acid	2004	SDG	Cocrystal	[155]
Caffeine or Theophylline + chiral or racemic forms D,L-tartaric acid	2006	LAG	Cocrystal	[311]
Ternary system Caffeine + Succinic acid + diverse solvent guest	2006	NG/LAG/slow evaporation	Cocrystal solvent	[312]
Caffeine or Theophylline + citric acid	2007	NG/LAG	Cocrystal	[212]
Theophylline + 1,7-Heptanediamine	2007	NG	Salt-Cocrystal Continuum	[61]
Nicotinamide + Mandelic acid or Ibuprofen	2007	NG/LAG	Cocrystal	[313]
Carbamazepine + diverse coformers	2009	SDG/slow evaporation	Cocrystal	[149]
Theophylline or Caffeine + L-Malic or L-Tartaric acid	2009	LAG/sonochemical reactions	Cocrystal	[206]
Meloxicam + Succinic acid or Maleic acid	2009	SDG	Cocrystal	[314]
Nicotinamide + 10 diverse dicarboxylic acids coformers	2009	Melt/NG/LAG/slow evaporation	Cocrystal	[241]
Paracetamol + 13 diverse coformers	2009	NG/LAG	Cocrystal	[306]
2-Chloro-4-nitrobenzoic acid + Nicotinamide	2010	LAG/slow evaporation	Cocrystal	[315]
Indomethacin + 30 diverse coformers	2011	LAG/Prediction of cocrystal formation employing Hansen solubility parameter	Cocrystal	[316]
Piracetam + Citric acid or Tartaric acid	2011	NG/LAG	Cocrystal	[317]
Nicotinamide + five Fenamic acid derivatives	2011	LAG/liquid assisted sonication/slow evaporation	Cocrystal	[318]
Curcumin + Resorcinol or Pyrogallol	2011	LAG	Cocrystal	[319]
Prulifloxacin + Salicylic acid	2011	Kneading	Cocrystal	[320]
Furosemide + 8 diverse coformers	2012	LAG	Cocrystal	[321]
Acetazolamide + diverse carboxilic acids or amide derivatives coformers	2012	LAG	Cocrystal	[322]
Indomethacin + Saccharin	2012	LAG	Cocrystal	[323]
Nitrofurantoin + 4-Hydroxybenzoic acid or Nicotinamide or L-Proline or Vanillic acid	2012	LAG	Cocrystal	[324]
Piroxicam + 20 different carboxylic acids	2012	LAG/fast cooling and slow cooling of a hot saturated solution/precipitation with an antisolvent/slow evaporation/melting	Cocrystal/cocrystal hydrate/coamorphous	[325]
Meloxicam + diverse carboxylic acids	2012	SDG	Cocrystal	[326]
DL-Malic acid + L-Tartaric acid and L-Malic acid + DL-Tartaric acid	2012	LAG	Cocrystal	[327]
Andrographolide (NP) + Vanilin or Vanillic acid or Salicylic acid or Guaiacol	2013	LAG	Cocrystal	[328]
α- or γ-Glycine + 7 carboxylic acids coformers	2013	NG/spray drying/fast and slow anti-solvent techniques	Cocrystal/multicomponent salt	[329]
Racemic Praziquantel + diverse aliphatic dicarboxylic acids coformers	2013	LAG/slurry method	Cocrystal	[330]
Carbamazepine + Saccharin and Nicotinamide + Suberic acid	2013	LAG	Cocrystal	[200]
L-Serine (anhydrous or monohydrate) + Oxalic acid (anhydrous or dihydrate)	2014	NG/LAG/slow evaporation/precipitation on antisolvent crystallization	Multicomponent salt/multicomponent salt hydrate/multicomponent salt polymorph	[331]
Trospium chloride + diverse carboxylic acid coformers	2014	NG/LAG/slurry method/slow evaporation	Cocrystal	[332]
AMG 517 + Sorbic acid	2014	Ball-milling/TSE	Cocrystal	[294]
Caffeine + Anthranilic acid	2014	LAG	Cocrystal polymorph	[68]
Lenalidomide + urea or 3,5-Dihydroxybenzoic acid (1:1 and 1:2:1 monohydrate)	2014	LAG	Cocrystal	[333]
Ezetimibe + Methyl paraben	2014	LAG/slow evaporation	Cocrystal	[334]
α-Glycine + β-Malonic acid	2014	NG/LAG/Impact treatment/shear treatment/vibratory treatment	Multicomponent salt	[335]
Stanozolol + Malonic acid or D-Phenyllactic acid or 6-Hydroxy-2-Naphthoic Acid	2014	LAG	Cocrystal	[336]
Caffeine + Citric acid or Anthranilic acid and Phenazine + Mesaconic acid	2015	LAG/POLAG	Cocrystal	[211]
Pyrazinamide + *p*-Nitrobenzoic acid	2015	LAG	Stoichiometric cocrystals	[337]
Theobromine + Oxalic acid	2015	NG/structure solved based on the powder X-ray data/in situ using synchrotron powder X-ray diffraction	Cocrystal	[338]
Theophylline + 4-Aminosalicylic acid or 4-Aminobenzoic acid	2015	LAG/slow evaporation	Cocrystal	[339]
Pyrimidin-2-amine + Glutaric acid	2015	NG/slow evaporation	Cocrystal/salt-cocrystal continuum	[340]
Theophylline + Benzoic acid	2015	NG/In Situ investigations of milling reactions using combined powder X-ray Diffraction and Raman spectroscopy	Cocrystal	[341]
Anthranilic acid + Carbamazepine or Salicylic acid or Theophylline and Salicylic acid + theobromine	2015	NG/LAG/slurry methods/competitive milling reactions	Cocrystal	[342]
Pentoxifylline + diverse carboxylic acid derivatives or Furosemide or L-Ascorbic acid	2015	NG/LAG/*In Silico* screening	Cocrystal	[343]
Triamterene + DL-Mandelic acid or Saccharin	2015	LAG/slurry method	Cocrystal	[344]
Theophylline + *o*-Aminobenzoic acid or *m*-Aminobenzoic acid or *p*-Aminobenzoic acid	2015	LAG/slow evaporation	Cocrystal	[345]
Adefovir Dipivoxil + Glutaric acid	2015	LAG	Cocrystal	[346]
Resveratrol + 4-Aminobenzamide or Isoniazid	2016	LAG/rapid solvent removal	Cocrystal	[307]
Ethionamide + Oxalic acid or Glutaric acid or Adipic acid or Sebacic acid or Fumaric acid	2016	LAG	Cocrystal/multicomponent salt	[347]
Piroxicam + Saccharin	2016	NG/LAG/slow evaporation	Cocrystal	[348]
Theophylline + Benzamide	2016	LAG/solvent screening/in situ synchrotron powder X-ray diffraction	Cocrystal/Cocrystal polymorph	[349]
Glicazide + Malic acid or Succinic acid	2016	LAG	Cocrystal	[350]
Ibuprofen + Nicotinamide	2016	NG/in situ Raman spectroscopy	Cocrystal	[351]
Simvastatin + Malic acid	2016	LAG	Cocrystal	[352]
Theophylline + Benzamide	2016	NG/synchrotron X-ray powder diffraction data	Cocrystal	[353]
Meloxicam + Acetylendicarboxylic acid	2016	LAG/slow evaporation	Cocrystal	[354]
Theophylline + Benzamide or Benzoic acid or Isonicotinamide	2016	NG/Competitive Cocrystal Reactions/in situ powder X-ray diffraction	Cocrystal	[355]
Pyrazinamide + Oxalic acid	2016	NG/LAG/in situ using combined synchrotron Powder X-ray Diffraction and Raman	Cocrystal	[356]
5-Fluorouracil + 3-Hydroxybenzoic acid or 4-Aminobenzoic acid or Cinnamic acid	2016	LAG/slurry method	Cocrystal	[357]
Lamotrigine + 4,4′-Bipyridine or 2,2′-Bipyridine	2017	LAG	Cocrystal	[358]
Aripiprazole + Orcinol	2017	LAG	Cocrystal	[359]
Hydrochlorothiazide + Piperazine or Tetramethylpyrazine or Picolinamide or Isoniazid or Malonamide or Isonicotinic acid	2017	NG/LAG	Cocrystal	[360]
Glicazide + Sebacic acid or α-Hydroxyacetic acid	2017	LAG	Cocrystal	[361]
Praziquantel + Citric acid or Malic acid or Salicylic acid or Tartaric acid	2017	NG/LAG	Cocrystal	[362]
γ-Glycine + Oxalic acid dihydrate	2017	NG/Real-time in situ X-ray powder diffraction	Multicomponent salt	[363]
Mycophenolic acid + Isonicotinamide or Minoxidil or 2,2′-Dipyridylamine	2017	LAG/slow evaporation	Cocrystal	[364]
Gliclazide + Catechol or Resorcinol or *p*-Toluene sulfonic acid or Piperazine	2017	LAG/slow evaporation	Cocrystal/multicomponent salt	[365]
Pyrazinamide + Malonic acid	2017	NG/LAG/slurry methods/In situ Powder X-ray Diffraction	Cocrystal polymorph	[366]
Carbamazepine + *p*-Aminosalicylic acid	2017	LAG/slurry methods/slow evaporation	Cocrystal	[367]
Theobromine + Oxalic acid and Pyrazinamide + Oxalic acid	2017	NG/LAG/in situ Raman experiments	Cocrystal	[368]
Felodipine + Imidazole	2017	LAG/in situ Raman experiments	Cocrystal	[369]
Metformin hydrochloride + Dehydrated disodium succinate	2017	NG/melting/slow evaporation	Multicomponent salt	[370]
Chlorothiazide + 13 diverse coformers	2017	LAG/slow evaporation	Cocrystal/multicomponent salt	[371]
11-Azaartemisinin + 13 diverse carboxylic acids coformers	2018	LAG	Cocrystal	[372]
Fluoxetine·HCl + Fumaric acid or Benzoic acid or Succinic acid	2018	NG/LAG/solvothermal synthesis/slow evaporation	Cocrystal	[373]
11-Azaartemisinin + *trans*-Cinnamic or Maleic acid (1:1 and 2:1) or Fumaric acid	2018	LAG	Cocrystal	[374]
Seselin (NP) + Thiourea	2018	LAG/slow evaporation	Cocrystal	[375]
β-Alanine + DL-Tartaric acid	2018	NG/LAG	Cocrystal/multicomponent salt	[258]
Naproxen + Proline	2018	LAG	Cocrystal polymorph/cocrystal hydrate/cocrystal solvate	[376]
Caffeine + Dapsone	2018	Slow evaporation/LAG/spray drying	Cocrystal	[377]
Theophylline + Aspirin	2018	NG/LAG/slurry method/ternary phase diagram	Multidrug cocrystal	[378]
Caffeine + Citric acid or Glutaric acid	2018	ILAG	Cocrystal polymorph	[217]
Glipizide + Glycolic acid	2018	NG/LAG/slurry method/slow evaporation	Cocrystal	[379]
Theophylline + Benzamide	2018	LAG	Cocrystal polymorph	[380]
Diclofenac acid + L-Proline	2018	NG/LAG	Cocrystal	[381]
Pefloxacin + 10 diverse dicarboxylic acids	2018	LAG/solvent evaporation	Multicomponent salt/multicomponent salt hydrate/salt cocrystal	[382]
Tofogliflozin + Sodium acetate or Potassium acetate	2018	LAG	Salt cocrystal	[383]
Flurbiprofen + Proline	2018	LAG/in situ Variable Temperature Synchrotron X-ray Diffraction	Chiral cocrystal/cocrystal solvate/stoichiometric cocrystal/cocrystal polymorph	[384]
Piroxicam + Succinic acid or Methylparaben or Resorcinol	2019	LAG	Cocrystal	[385]
Lamotrigine + Phthalimide or Succinimide	2019	LAG/slow evaporation/Ternary phase diagram	Cocrystal/Cocrystal hydrate	[386]
Pyrazinamide + Pimelic acid	2019	NG/LAG/Time Resolved In situ Powder X-ray Diffraction	Cocrystal polymorph	[231]
Nevirapine + *p*-Aminobenzoic acid	2019	NG/LAG	Cocrystal	[387]
Luteolin (NP) + Isoniazid or Caffeine	2019	LAG/Rapid solvent removal	Cocrystal	[388]
Meloxicam + Salicylic acid or Fumaric acid or Malic acid	2019	LAG	Cocrystal	[389]
Oxcarbazepine + Oxalic acid or 2,5-Dihydroxybenzoic acid or Salicylic acid	2019	LAG/slow evaporation	Cocrystal	[390]
α-D-Glucose + NaCl or NaBr or NaI	2019	NG/LAG	Ionic cocrystal	[391]
Carbamazepine + DL-Mandelic acid or DL-Tartaric acid	2019	LAG/computational prediction	Cocrystal/cocrystal polymorph	[392]
2-Pyridine-carboxaldehyde benzoylhydrazone (hydrazone) + Malonic acid + Succinic acid + Glutaric acid + Mesaconic acid	2019	NG/LAG/slow evaporation	Cocrystal/cocrystal solvate/multicomponent salt	[393]
Betulin + Adipic acid or Succinic acid or Suberic acid	2019	LAG	Cocrystal	[394]
Ciprofloxacin + Salicylic acid	2019	LAG/in situ Raman spectroscopy experiments	Multicomponent salt/multicomponent salt hydrate and solvate/salt-cocrystal	[395]
Pyrazinamide + Glutaric acid + Isonicotinamide and Pyrazin-2-carboxylic acid + Glutaric acid + Isonicotinamide	2019	LAG/In situ Powder X-ray Diffraction	Ternary cocristal	[396]
Pirfenidone + Fumaric acid or Trimesic acid	2019	LAG/slow evaporation	Cocrystal	[397]
Glipizide + Glutaric acid	2019	NG/LAG/slow evaporation/slurry method	Cocrystal	[398]
Gemfibrozil + Isonicotinamide	2019	Milling	Cocrystal	[399]
Salicylic acid + diverse Imidazole coformers	2019	NG/structures were solved by powder X-ray diffraction	Multicomponent salt	[400]
Caffeine + Glutaric acid	2020	NG/LAG/POLAG	Cocrystal	[214]
Oxyresveratrol + Nicotinamide or Proline	2020	LAG/employing principal component analysis	Cocrystal	[401]
Itraconazole + Terephtalic acid	2020	LAG	Cocrystal	[402]
Ciprofloxacin + Nicotinic acid or Isonicotinic acid	2020	LAG	Cocrystal	[403]
Nicotinamide + Adipic acid	2020	Ball-milling LAG/Demonstration of reversible mechanochemical cocrystal polymorph interconversion (stable → metastable phase transformation). This process can be controlled by the choice of milling assembly/Real-time X-ray powder diffraction	Cocrystal polymorph	[229]
Betulin + Terephthalic acid	2020	LAG	Cocrystal	[404]
Ciprofloxacin + Carvacrol or Thymol	2020	NG/ball-milling LAG/slow evaporation/slurry method	Cocrystal	[405]
5-Fluorouracil + Kaempferol	2020	LAG/slurry method/slow evaporation/ternary phase diagram	Multidrug cocrystal	[406]
Chromotropic acid + 1,10-Phenanthroline	2020	LAG/solvent evaporation	Cocrystal salt hydrate	[407]
Ibuprofen + Nicotinamide	2020	Ball-milling NG/melting/slow evaporation. Detection of cocrystal polymorphism (2 forms). Formation of one or another polymorph depend on the synthetic method used.	Cocrystal polymorph	[243]
Thiobarbituric acid or Barbituric acid + 1-Hydroxy-4,5-Dimethyl-Imidazole 3-Oxide	2020	LAG/solvent evaporation	Cocrystal	[408]
Telmisartan + Hydroclorothiazide	2020	LAG/slow evaporation	Multidrug cocrystal	[409]
Chlorothiazide + 13 diverse coformers	2020	Ball-milling LAG/Study of the effect of grinding on 11 cocrystals an one salt in the presence of the excipients polyvinylpyrrolidone and microcrystalline cellulose	Cocrystal/multicomponent drug	[410]
Nebivolol hydrochloride + 4-Hydroxybenzoic acid or Nicotinamide	2020	LAG	Cocrystal	[411]
9-Ethyladenine + Malonic acid or Succinic acid or Fumaric acid or Glutaric acid or Adipic acid	2020	LAG/slow evaporation	Cocrystal/multicomponent salt	[412]
Carbamazepine + DL-Tartaric acid	2020	LAG	Cocrystal	[413]
Emtricitabine + 1,2-Bis(4-pyridyl)ethane or 1,2-Bis(4-pyridyl)ethylene or 4,4′-Azopyridine or 4,4′-Bipyridine	2020	LAG	Cocrystal	[414]
Caffeine + Glutaric acid	2020	NG/LAG/POLAG/in situ X-ray powder diffraction	Cocrystal	[214]
Cocrystal (Barbituric acid/Thiobarbituric acid) BA_0_._5_TBA_0_._5_ + 1-Hydroxy-4,5-dimethyl-imidazole 3-oxide	2020	Ball-milling LAG/Preparation of ternary cocrystal system beginning from binary cocrystal system. Evaluation of the possible pathways involved, the evidence suggests a non-concerted process.	Binary and ternary cocrystal	[245]
Tinidazol + *p*-Aminobenzoic acid or Citric acid or Salicylic acid	2020	NG/LAG	Cocrystal	[415]
Ciprofloxacin + Pyrazinoic acid or *p*-Aminobenzoic acid	2020	NG/LAG	Cocrystal	[416]
Zaltoprofen + Nicotinamide (1:1 or 1:2)	2020	LAG	Cocrystal	[417]
Metronidazole + 3,5-Dihydroxybenzoic or 3,4,5-Trihydroxybenzoic acid	2020	LAG/melt/slow evaporation	Cocrystal	[418]
Exemestane + 9-Hydroxyphenanthrene and 1-Hydroxypyrene	2020	LAG	Cocrystal	[419]
Penciclovir + 3,5-Dihydroxybenzoic acid or Gallic acid (1:1 or 1:1:1 hydrate) or 4-Hydroxycinnamic acid (1:1 or 1:1:1 hydrate)	2020	LAG	Cocrystal/cocrystal hydrate	[420]
Berberine chloride + Pyromellitic dianhydride	2020	LAG	Diverse multicomponent stoichiomorphs: multicomponent salt/multicomponent salt polymorph/ionic cocrystal hydrate	[421]
Allopurinol + Isonicotinamide or Piperazine or 2,4-Dihydroxybenzoic acid	2020	LAG/slurry methods/slow evaporation	Cocrystal	[422]
Trimethoprim + Flufenamic acid or Tolfenamic acid or Mefenamic acid and Sulfamethazine + Flufenamic acid or Niflumic acid	2020	LAG/slow evaporation	Multicomponent salt hydrate/multidrug cocrystal	[423]
Ciprofloxacin + 4-Hydroxybenzoic acid or 4-Aminobenzoic acid or Gallic acid	2020	LAG	Multicomponent salt hydrate	[424]
Regorafenib + Malonic acid or Glutaric acid or Pimelic acid	2021	LAG/slurry methods	Cocrystal	[425]
Theobromine + Trimesic acid or Hemimellitic acid and Caffeine + Trimesic acid or Hemimellitic acid	2021	NG/LAG/slow evaporation	Cocrystal/multicomponent salt/cocrystal hydrate	[426]
Temozolomide + Hesperetin	2021	LAG/slurry methods/slow evaporation	Multidrug cocrystal	[427]

## Data Availability

No new data were created or analyzed in this study. Data sharing is not applicable to this article.

## Abbreviations

adi	Adipic acid
ana	Anthranilic Acid
API	Active Pharmaceutical Ingredient
α-alanine	α-ala
BA	Barbituric acid
BSC	Biopharmaceutics classification system
4,4′-bipy	4,4′-Bipyridine
ca	Citric Acid
caf	Caffeine
cbz	Carbamazepine
CSD	Cambridge structural database
CTA	Cyclohexane-1,3-*cis*-5-*cis*-tricarboxylic Acid
δ	δ parameter
dabco	1,4-diazabicyclo [2.2.2]octane
DL-ta	DL-tartaric acid
DSC	Differential Scanning Calorimetry
DTA	Differential thermal analysis
FDA	Food and drug administration
FT-IR	Fourier Transform Infrared Spectroscopy
GRAS	Generally recognized as safe
Glu	Glutaric acid
HIMO	4,5-dimethyl-imidazole-3-oxide
IL	Imidazolium based ionic liquids
ILAG	Ion liquid-assisted grinding
IUPAC	International Union of Pure and Applied Chemistry
LAG	Liquid-Assisted Grinding
LASI	Liquid-assisted sonochemical irradiation
ma	Mesaconic Acid
MeNO_3_	Methyl nitrate
MOF	Metal-organic framework
*m*	weights of cocrystal components
NP	Natural product
NG	Neat Grinding
Nicotinamide	nic
5-nip	5-Nitroisophtalic acid
η	η parameter
ox	Oxalic Acid
PEG	Polyethylene Glycol
phe	Phenazide
4,7-*phen*	4,7-phenanthroline
POLAG	Polymer-Assisted Grinding
PMMA	Poly(methyl methacrylate)
PXRD	Powder X-ray Diffraction
RAM	Resonant acoustic mixing
RS-ibp	RS-ibuprofen
sa	Salicylic Acid
SCXRD	Single Crystal X-ray Diffraction
SDG	Solvent Drop Grinding
S-ibp	S-ibuprofen
ssNMR	solid-state Nuclear Magnetic Resonance
ta	Terephthalate
thp	Theophillyne
TBA	Thiobarbituric acid
TGA	Thermogravimetric Analysis
TSE	Twin-screw extrusion
tp	Theophylline
*V*	volume
VALAG	Variable amount liquid-assisted grinding
VATEG	Variable temperature grinding
XPS	X-ray photoelectron spectroscopy
